# Immunostimulatory Phosphatidylmonogalactosyldiacylglycerols (PGDG) from the Marine Diatom *Thalassiosira weissflogii*: Inspiration for a Novel Synthetic Toll-Like Receptor 4 Agonist

**DOI:** 10.3390/md17020103

**Published:** 2019-02-09

**Authors:** Emiliano Manzo, Carmela Gallo, Rossella Sartorius, Genoveffa Nuzzo, Angela Sardo, Piergiuseppe De Berardinis, Angelo Fontana, Adele Cutignano

**Affiliations:** 1 CNR-Institute of Biomolecular Chemistry, via Campi Flegrei, 34, 80078 Pozzuoli (Napoli), Italy; emanzo@icb.cnr.it (E.M.); carmen.gallo@icb.cnr.it (C.G.); nuzzo.genoveffa@icb.cnr.it (G.N.); angela.sardo@icb.cnr.it (A.S.); afontana@icb.cnr.it (A.F.); 2 CNR-Institute of Protein Biochemistry, via Pietro Castellino, 111, 80131 Napoli, Italy; r.sartorius@ibp.cnr.it (R.S.); p.deberardinis@ibp.cnr.it (P.D.B.)

**Keywords:** marine diatom, *Thalassiosira weissflogii*, phosphogalactolipids, Toll-like receptor 4, dendritic cells, immunostimulatory lipid, phosphoglycolipid synthesis

## Abstract

An unprecedented phosphatidylmonogalactosyldiacylglycerol pool (PGDG, **1**) rich in polyunsaturated fatty acids was isolated from the marine diatoms *Thalassiosira weissflogii*. Here we report for the first time the NMR characterization of this rare lipid from marine organisms along with a synthetic strategy for the preparation of a PGDG analog (**2**). PGDG **1** exhibited immunostimulatory activity in human dendritic cells (DCs) and the synthetic PGDG **2** was prepared to explore its mechanism of action. A Toll-like receptor-4 (TLR-4) agonistic activity was evidenced in human and murine DCs underlying the antigen-specific T-cell activation of this class of molecules.

## 1. Introduction

Glycerolipids containing both a carbohydrate and a phosphate group as structural components are uncommon in nature. They have been classified in two groups named ‘glycophospholipids’ and ‘phosphoglycolipids’, respectively. Members of the first group include phosphatidylglucosides reported from bacterial and, more recently, from mammalian sources [1,2]. They are typically characterized by saturated fatty acids and are minor lipids in quantitative terms although they are thought to play important biological functions [3,4]. On the other hand, phosphoglycolipids are typical bacterial products, for the first time identified in the 1960s [5] and afterwards characterized only by chemical degradation [6,7,8,9,10,11] and mass spectrometric techniques [12]. In phosphoglycolipids, the phosphate moiety is located on position 6 of the sugar while in glycophospholipids typically occurs on the anomeric carbon 1. Many glycolipids exhibited immunomodulatory functions as previously reported [13,14,15] and immunostimulants represent an emerging class of drugs for the treatment of infectious disorders and cancer. In the frame of a biological screening of marine extracts on Peripheral Blood Mononuclear Cells (PBMC) from human donors, the diatom *Thalassiosira weissflogii* (CCMP 1336) was selected due to its immunostimulatory properties, measured as production of IL-6, a cytokine with pleiotropic activity in immune regulation, hematopoiesis, inflammation and oncogenesis [16]. A bioassay guided fractionation of the active extract on LH-20 resin afforded a fraction featured by low abundance polar lipid species exhibiting a molecular weight (MW) between 1300 and 1600 amu in HR-ESI^−^MS. This mass range was significantly higher than MW of major glycerol-based lipids typically reported from *T. weissflogii* as well as from other microalgae and prompted us to further investigation [17,18,19]. Here we report molecular characterization, synthesis of a structural analog and immunostimulatory properties of a novel pool of phosphoglycolipids originally isolated from this marine diatom.

## 2. Results and Discussion

**Figure 1:**
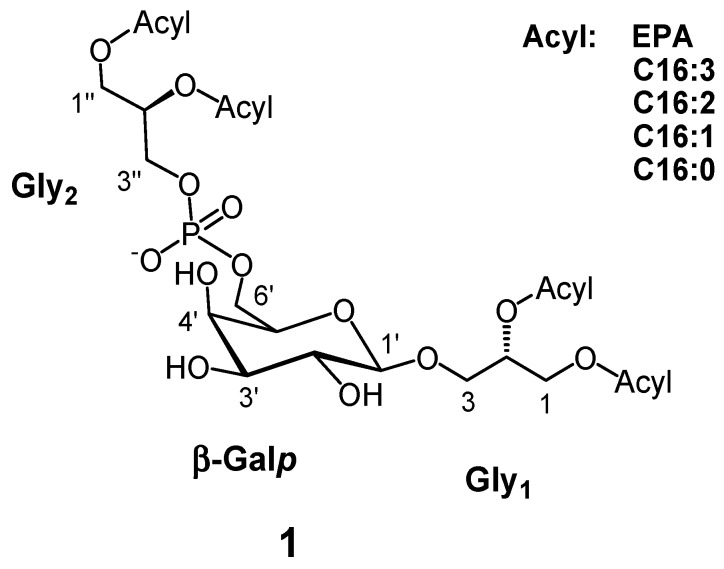
Core structure of PGDG **1** from *Thalassiora weissflogii*. Main acyl chains are reported.

Preliminary NMR data on the bioactive LH-20 fraction from *T. weissflogii* extract suggested the presence of a monoglycoglycerolipid backbone. A further purification step by HPLC was carried out and a homogeneous bioactive fraction was subjected to spectroscopic (NMR) and spectrometric (ESI-MS/MS) analyses. Spectral data, along with information from chemical degradation allowed to characterize the complex pool as unusual phosphatidylmonogalactosyldiacylglycerols (PGDG, **1**, Figure 1). 

Structural characterization of this class of lipids was not straightforward due to the minute amount recovered and severe overlapping of proton NMR signals in the region between δ 3.5 and 4.2 of the two glycerols and the sugar protons along with lack of corresponding NMR data reference in the literature for this class of lipids. The monosaccharide residue was identified by both NMR data (Table 1) and acidic degradation as a β-galactopyranose. In fact, by 2D NMR experiments an extended spin system was reconstructed, which connected the anomeric proton at δ 4.23 (C1′, 104.5 ppm) to methine protons at δ 3.53 (C2′, 71.9 ppm), 3.51 (C3′, 73.7 ppm), 3.99 (C4′, 68.3 ppm), 3.63 (C5′, 74.2 ppm) and to methylene at 4.09/3.92 (C6′, 63.4 ppm). In particular, the coupling constants *J* = 9.7 and 3.3 Hz measured for proton H-3′, were indicative of axial/axial and axial/equatorial orientations of the vicinal protons H2′/H3′ and H3′/H4′, respectively. Furthermore the coupling constant between H-1′ and H-2′ (*J*_1,2_ = 7.2 Hz) along with the chemical shift of the carbon C1′ (104.5 ppm) suggested a β-configuration of the anomeric carbon of the sugar which was therefore assigned as β-galactopyranose. Its identity was confirmed by ion exchange chromatography after TFA hydrolysis, in comparison with authentic standards. The anomeric carbon at 104.5 ppm showed HMBC connection through a glycosidic bond with the methylene protons of a glycerol residue at δ 3.99 and 3.68 (Gly_1_-C3, 68.0 ppm) which in turn were coupled with the oxymethine proton at δ 5.25 (Gly_1_-C2, 71.0 ppm) and the oxymethylene protons at δ 4.40 and 4.23 (Gly_1_-C1, 63.3 ppm). The downfield chemical shifts of the glycerol protons at C1 and C2 were in agreement with the presence of acyls at these positions.

**Figure 2:**
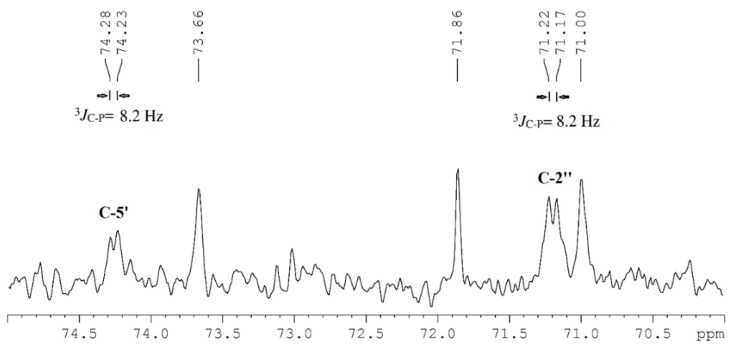
^13^C NMR spectrum enlargement of **1** showing C-P couplings.

However, HSQC data indicated the presence of another glycerol residue (Gly_2_), whose protons resonated at almost the same values of Gly_1_ but distinguishable by the carbon chemical shifts values at 63.4 (Gly_2_-C1″), 71.2 (Gly_2_-C2″) and 64.1 ppm (Gly_2_-C3″). In particular, the ^13^C NMR signal centered at 71.2 ppm appeared clearly split in a doublet with a constant of 8.2 Hz likely due to a ^3^*J*_C-P_ coupling (Figure 2) [20]. Another doublet was observed for carbon C5′ of the galactose residue at 74.3 ppm (^3^*J*_C-P_ = 8.2 Hz) thus suggesting that the second glycerol moiety was joined through a phosphate group at Gly_2_-C3𠌣 to the C6′ of the sugar (Figure 2). As a confirmation, signal at δ 4.09 (H-6′a), resonating in a non-crowded region of the protonic spectrum, showed a complex multiplicity due to ^3^*J*_H-P_ coupling (Supporting Information, Figure S1) [21]. 

According to deshielded ^1^H and ^13^C NMR resonances (Table 1) and LC-ESI^-^MS/MS analysis, both glycerols were 1,2-acylated by fatty acids, whose relative composition was inferred by GC-MS analysis of the hydrolyzed PGDG pool (Supporting Information, Table S1). Furthermore, detailed fatty acids (FA) composition of main lipid species was inferred by extensive HR-ESI^−^MS/MS analysis (Supporting Information, Figure S19 and Table S2). The natural PGDG pool resulted mainly composed by unsaturated fatty acids, including eicosapentaenoic acid (EPA) and analogs from the C16 series, mainly C16:3 and C16:2.

**Scheme 1:**
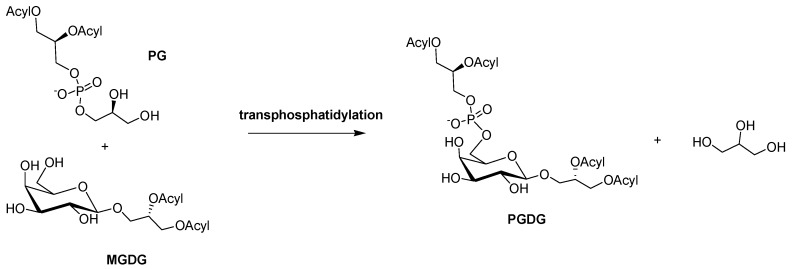
Possible biogenetic origin of phosphatidylmonogalactosyldiacylglycerol (PGDG) from monogalactosyldiacylglycerol (MGDG) and phosphatidylglycerol (PG) by trans-phosphatidylation.

As put forward for bacterial phosphatidylmonoglucosylglycerides, the origin of PGDG lipids can be biosynthetically traced back to a transphosphatidylation reaction between a phosphatyl group donor such as phosphatidylglycerol (PG) and a monogalactosyldiacylglycerol (MGDG) [22,23]. Indeed, the fatty acid profile of PGDG **1** (Supporting Information, Table S2) matches that of MGDG and PG from marine diatoms and in particular from this species as already reported in our previous paper, both highly rich in polyunsaturated fatty acids [17,18,19]. Following this line of reasoning, both glycerol moieties should belong to the 1,2-diacyl-3-X-*sn*-glycerol series, reflecting the stereochemistry of the biosynthetic precursors as depicted in Scheme 1. The fatty acid position on 1,2-glycerols could not be established in our LC-MS conditions due to existence of isobaric species and poor chromatographic resolution, which hampered specific fragmentation studies. Nevertheless, the plastidial origin of both MGDG and PG precursors typically carrying C16 fatty acids at position 2 and EPA at position 1 of glycerol, as previously reported [17], suggests the same fatty acid relative distribution on position 1 and 2 of glycerol moieties in PGDG lipids.

In the literature, there are very few examples of bacterial galactose-based phosphoglycolipids [10] and a unique report from marine bacteria of a glucose analog [12]; in all cases, these lipids bear saturated fatty acids and have been characterized only by mass spectrometry and degradative chemistry. Here we document the first finding, and provide full NMR structural data, of galactopyranose-based phosphoglycolipids, decorated with polyunsaturated fatty acids, from marine environment. 

PGDG are very minor lipids in *T. weissflogii* compared to other related galactolipids like MGDG but also with respect to other lipid classes, such as sulfoquinovosides, phospholipids and triacylglycerols [17,24]. The biological function of PGDG in diatoms is still unknown: a structural role in membranes or as storage lipids seems unlikely due to their low abundance, but cannot be ruled out. Interestingly, we found an increased amount of these lipids in the late stationary-initial senescent phase of the growth curve with respect to exponential/stationary phase (data not shown). Different levels of metabolites during the cell growth may have a biological significance, implying for example a signaling or regulatory role, or a stress response. The latter aspect is particularly invoked when an increase of metabolite levels is observed in coincidence with reduced availability of nutrients as occurs in the late phase of a culture growth. This observation opens intriguing perspectives to further biological studies aiming at explore the PGDG function. 

**Figure 3:**
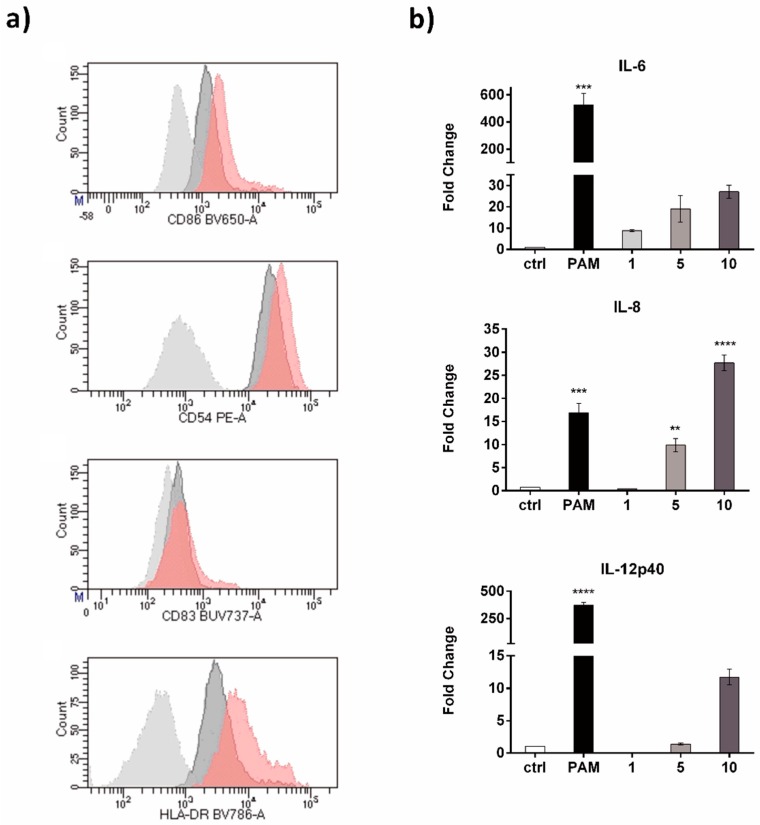
(**a**) Phenotypic surface markers analysis of monocyte-derived dendritic cells (moDCs): isotypic control (light grey), control cells (ctrl) (dark grey), treated cells with PGDG (**1**) (pink); (**b**) Cytokine gene expression upon stimulation of moDCs with PAM2CSK4 (PAM, 1 µg/mL) as positive control, with natural PGDG (**1**) at concentrations of 1, 5 and 10 µg/mL compared to cells treated only with vehicle (ctrl). Data are expressed as fold change +/− SD after 24 h exposition. One representative experiment of two is shown. Asterisks indicate significant differences from ctrl; *p* < 0.001 by one-way ANOVA and the Tukey multiple comparison test. ** *p* < 0.01, *** *p* < 0.001, ^****^ *p* < 0.0001.

In order to characterize the immunological properties of PGDG **1** we used an in vitro tool based on monocyte-derived dendritic cells (moDCs) [25]. DCs are potent innate immune cells and represent highly specialized antigen-presenting cells (APC) that play pivotal roles in the induction of protective immunity against potentially harmful antigens [26]. DCs are characterized by an “immature” state followed by maturation upon activation [26]. Maturation of moDCs was assessed by measuring the expression of major histocompatibility complex (MHC)-Class II (HLA-DR), intercellular adhesion molecule (ICAM, CD54), and the co-stimulatory molecules CD83 and CD86 by flow cytometry [27,28]. The bioactive fraction from *T. weissflogii* extract was tested on moDCs in the range of 10–50 µg/mL and showed a saturating effect on upregulation of surface markers already at the lowest concentration (Figure S20). We therefore selected 1–10 µg/mL as the best range to test the purified PGDG pool. When assayed on human moDCs in this range, PGDG **1** displayed immunostimulatory activity at 10 µg/mL inducing moDCs maturation (Figure 3a). All the phenotypic markers were upregulated after treatment with PGDG **1**. The compounds also elicited the mRNA overexpression of interleukins IL-6, IL-8 and IL-12p40 from 5 µg/mL (Figure 3b). These results are not totally surprising since glycoconjugates and in particular glycolipids are involved in cell-cell recognition mechanisms and immunological response [29,30,31,32]. 

**Figure 4:**
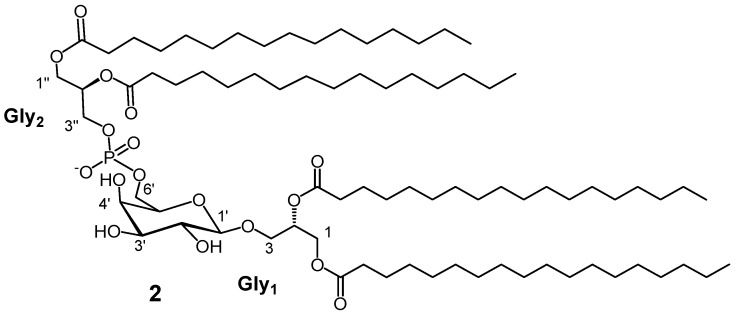
Chemical structure of synthetic PGDG **2**.

**Scheme 2:**
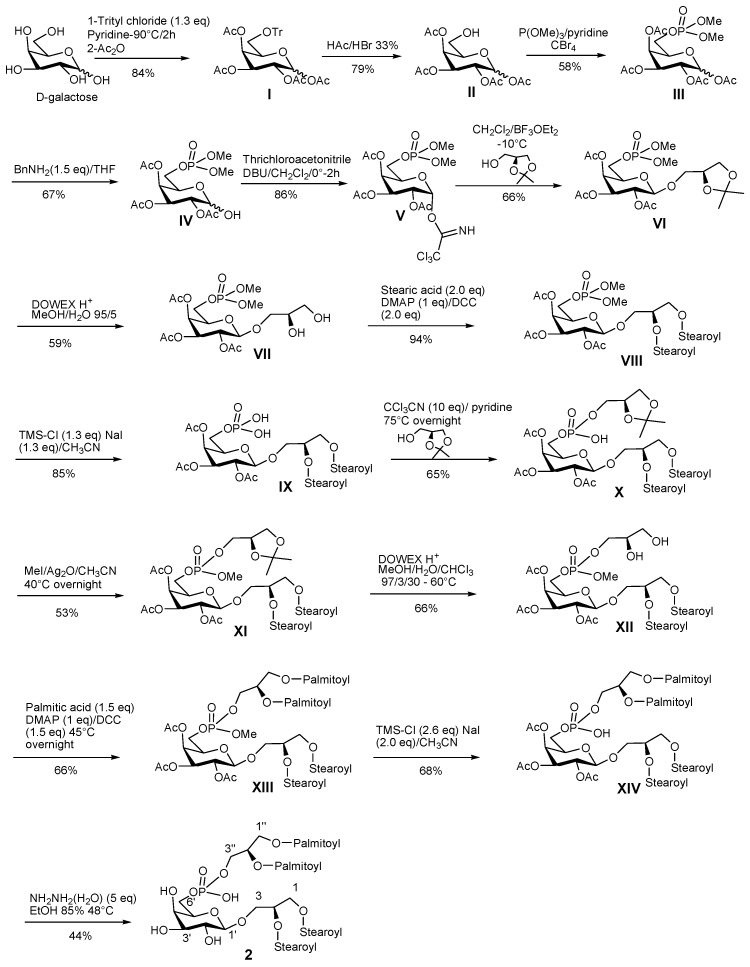
Synthetic procedure for 3-*O*-[6′-*O*-(1″,2″-dipalmitoyl-3″-phosphoryl-*sn*-glycerol)-1′-β-d-galactopyranosyl]-1,2-distearoyl-*sn*-glycerol (**2**).

With the aim of exploring the molecular target and to get more insight into the immunological potential of PGDGs, considering the low amount and the inherent instability of these natural products, we developed a synthetic strategy for the preparation of a structural analog with two stearoyl and two palmitoyl fatty acids onto the Gly_1_ and Gly_2_ glycerol subunits, respectively (**2**, Figure 4 and Scheme 2). 

To the best of our knowledge only one chemical synthesis of phosphatidyl-β-glycosyl-diacylglycerol was developed by Van Boeckel et al. [33] in the 80s; in the reported procedure the β-glycosidic bond was formed by Koenigs-Knorr conditions using glucopyranosyl-bromide as precursor and mercuric salts as catalysts, but problems linked to the stability of one intermediate forced the authors to make the strategy longer and complicate. Here we adopted a new and versatile strategy for the preparation of **2** (Scheme 2). In particular, the phosphate group in 6′ position of the sugar unit was introduced by trimethyl phosphite/carbon tetrabromide reaction of 1′,2′,3′,4′-tetra-*O*-acetyl-galactose (**II**), prepared from commercial D-galactose; after deacetylation at anomeric carbon of **III** with benzylamine and preparation of trichloroacetimidate derivative (**V**), the coupling with (*S*)-1,2-*O*-isopropylidene glycerol aglycon led to 6′-*O*-dimethylphosphoryl-1′-β-d-2′,3′,4′-triacetyl-galactopyranosyl-*sn*-isopropylidene-glycerol (**VI**); the choice of the acetate as hydroxyl protecting group favored the formation of β-glycosidic linkage [34,35,36]. Hydrolysis of acetonide, condensation with stearic acid and demethylation of phosphate group with trimethyl-silyl-chloride led to 6′-*O*-phosphoryl-1′-β-d-2′,3′,4′-triacetyl-galactopyranosyl-1,2-distearoyl-*sn*-glycerol (**IX**) then functionalized on phosphate group with (*S*)-1,2-*O*-isopropylidene glycerol by phosphate trichloroacetimidate activation to get 3-*O*-[6′-*O*-(3″-phosphoryl-*sn*-isopropylidene-glycerol)-1′-β-D-2′,3′,4′-triacetyl-galactopyranosyl]-1,2-distearoyl-*sn*-glycerol (**X**), subsequently methylated at phosphate (**XI**). Deprotection of acetonide and condensation with palmitic acid gave 3-*O*-[6′-*O*-(1″,2″-dipalmitoyl-3″-methylphosphoryl-*sn*-glycerol)-1′-β-D-2′,3′,4′-triacetyl-galactopyranosyl]-1,2-distearoyl-*sn*-glycerol (**XIII**) that was finally de-*O*-methylated by trimethyl-silyl-chloride and deacetylated by hydrazine monohydrate [36] obtaining 3-*O*-[6′-O-(1″,2″-dipalmitoyl-3″-phosphoryl-*sn*-glycerol)-1′-β-d-galactopyranosyl]-1,2-distearoyl-*sn*-glycerol (**2**). NMR data of the final synthetic product were superimposable to those of **1** (Table 1), thus corroborating the proposed core structure together with glycerol moieties stereochemistry of **1**. 

**Figure 5:**
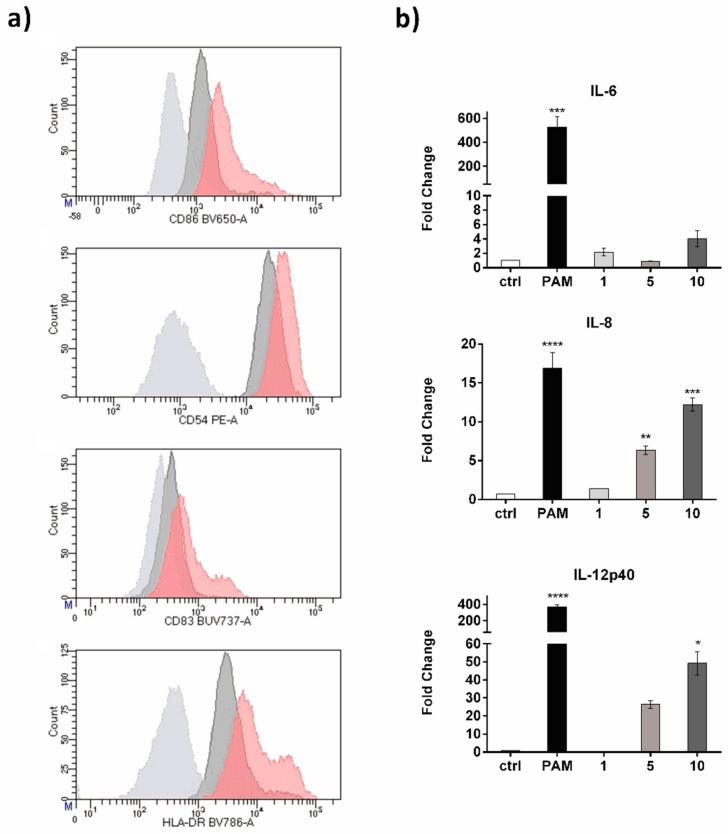
(**a**) Phenotypic surface markers analysis of moDCs: isotypic control (light grey), control cells (dark grey), treated cells with PGDG (**2**) (pink); (**b**) Cytokine gene expression upon stimulation of moDCs with PAM2CSK4 (PAM, 1 µg/mL) as positive control, with synthetic PGDG (**2**) at concentrations of 1, 5 and 10 µg/mL compared to cells treated only with vehicle (ctrl). Data are expressed as fold change +/− SD after 24 h exposition. One representative experiment of two is shown. Asterisks indicate significant differences from the ctrl; *p* < 0.001 by one-way ANOVA and the Tukey multiple comparison test. * *p* < 0.5, ** *p* < 0.01, *** *p* < 0.001, **** *p* < 0.0001.

We tested the activity of synthetic PGDG **2** on moDCs cells and we actually found an increase of the effect on cells maturation at 10 µg/mL. The synthetic compound elicited a more remarkable effect on the upregulation of characteristic moDCs markers, in particular on the co-stimulatory molecules CD83 and CD86 (Figure 5a) as well as on IL12p40 gene expression although there was a slight reduction on IL-6 and IL-8 expression with respect to the natural pool **1** (Figure 5b). In general, upon activation of moDCs, the production of IL-8 and IL-6 shows a faster kinetic rate than IL-12 and the up-regulation of co-stimulatory molecules, and can be already observed after 2 h from stimulation [37,38,39]. We may interpret the different expression of IL-6 and IL-8 induced by the two stimulants **1** and **2** as the result of different kinetics, with a faster, more potent effect of the synthetic analog **2**, acting earlier in the moDCs assay with a consequent anticipated decline, as observed at 24 h, of the expression level of the above interleukins compared to natural PGDG **1**. The differences in the stimulation profile could be due to the saturated acyl residues in PGDG **2** which may modulate inflammatory gene expression in moDCs to a different extent with respect to unsaturated acyl fatty acids of PGDG **1** and/or to different molecular targets involved in the mechanism of immunostimulation [39]. 

The effect of many immunostimulant lipids exhibiting phosphate groups on acylated aminosugars in their molecular architecture is mediated by Toll-like receptors (TLRs) [40,41]. Among these lipids, bacterial lipopolysaccharide (LPS) and its synthetic derivative monophosphoryl lipid A (MPLA) are the most widely studied agonists of TLR-4 [42]. Glycerol-based lipids with phosphate and carbohydrate in their structure represent another group of lipids for which an immunomodulant activity has been analyzed, even though only in a limited number of cases [13,15,43,44]. The best-known member of this group is lipotheicoic acid (LTA), composed by a glycoglycerolipid bound to a hydrophilic polymer of glycerophosphate, for which a specific TLR-2 agonistic activity has been documented [13,44]. 

**Figure 6:**
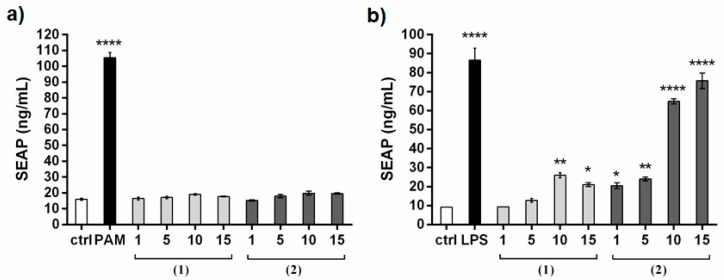
(**a**) TLR-2 NF-kB/SEAP reporter cell line assay with increasing concentrations of natural PGDG **1** and synthetic PGDG **2**; PAM2CSK4 (PAM) was used as positive control; (**b**) TLR-4 activation by increasing concentrations of natural PGDG **1** and synthetic PGDG **2** in TLR-4 NF-kB/SEAP reporter cell lines; LPS was used as positive control. Asterisks indicate significant differences from the cells treated only with vehicle (ctrl); *p* < 0.001 by one-way ANOVA and the Tukey multiple comparison test. * *p* < 0.05, ** *p* < 0.01, **** *p* < 0.0001.

To verify whether PGDG **1** and **2** activity were TLR-2/TLR-4 mediated, we tested their effects in a cell-based assay with HEK-293 cells stably co-transfected to express full-length human TLR-2 or TLR-4 and the secreted alkaline phosphatase (SEAP) reporter gene under the transcriptional control of a NF-kB response element [45]. The activity of these compounds is independent from TLR-2 induction. In fact, when both molecules were assayed for TLR-2 linked NF-kB SEAP activity, results were negative (Figure 6a). On the other hand, as shown in Figure 6b, natural PGDG **1** activated TLR-4 reporter cells at concentrations that stimulate maturation and cytokine expression in moDCs (10 µg/mL). Synthetic PGDG **2** has a comparable effect at the lowest concentrations, but doubled the SEAP production at 10 µg/mL.

**Figure 7:**
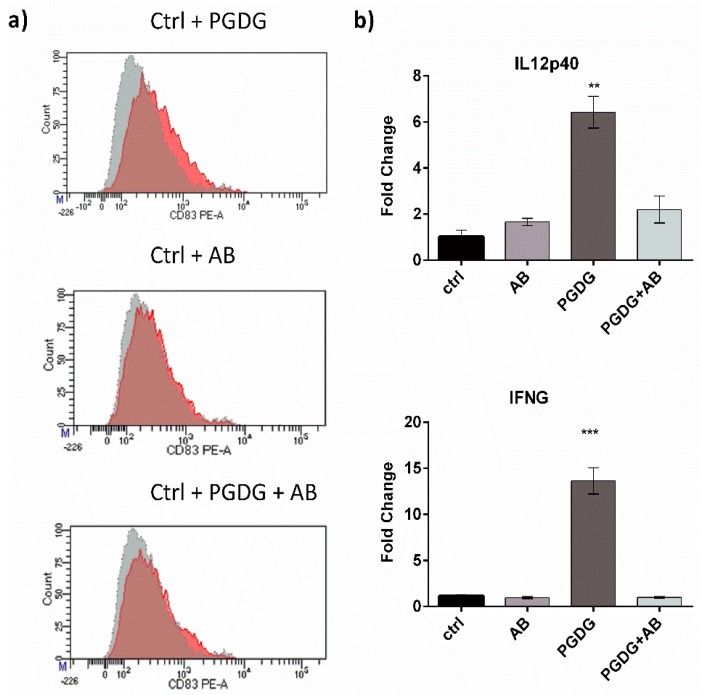
(**a**) moDCs CD83 surface expression: control cells (grey), treated cells (pink); (**b**) cytokine gene expression upon stimulation of moDCs with PGDG **2** at 10 µg/mL or with ab anti-TLR4 (AB) at 1 µg/mL, compared to the cells treated only with vehicle (ctrl). Data are expressed as fold change +/− SD after 24 h exposition. One representative experiment of two is shown. Asterisks indicate significant differences from the cells treated only with vehicle (ctrl); *p* < 0.001 by one-way ANOVA and the Tukey multiple comparison test. ** *p* < 0.01, *** *p* < 0.001.

To confirm the involvement of TLR-4 receptor, we applied a neutralizing antibody to block TLR-4 on moDCs. We used an anti TLR-4 antibody previously reported as blocking agent in in vitro experiments [46], to evaluate the effect after treatment with synthetic PGDG **2** by measuring CD83 on moDCs surface. High expression of this marker is a peculiarity of fully matured human DCs and critical for the in vivo priming of naïve T cells [47]. The concentration of 1 µg/mL was estimated as the dose of the antibody to cover all TLR-4 sites on moDCs surface. As shown in Figure 7a, in comparison with the stimulation with the phosphoglycolipid alone, CD83 surface expression of cells stimulated with PGDG **2** after treatment with TLR-4 antibody is nearly null. The residual effect could be due to partial blockage or to other mechanisms involving PGDG immunostimulation. Therefore we evaluated the upregulation of IL-12p40 and IFN-γ gene expression as more characteristic cytokines of DCs activation after TLR ligation [48]. The overexpression of both mediators disappeared in cells treated with PGDG and TLR-4 antibody (Figure 7b). 

Antigen presentation function of DCs is tightly regulated by the maturation process. A functional assay was set up to investigate the immunological properties of **2** in improving DCs antigen presentation and T cell activation. To this aim, mouse bone marrow-derived dendritic cells (BM-DCs) were incubated for 3 h with the synthetic peptide OVA_257–264_ in presence of **2** or LPS or MPLA as maturation stimuli. DCs were then assessed for their capability to triggering activation of OTI (OVA_257–264_ specific) hybridoma T cells B3Z, by evaluating interleukin-2 (IL-2) release in the culture supernatants in response to OVA_257–264_. B3Z CD8+ T cell line is known to produce high levels of IL-2 exclusively upon activation of T cell receptor specific for the OVA_257–264_ SIINFEKL peptide in the context of H-2Kb MHC class I complex expressed on antigen presenting cells [49].

**Figure 8:**
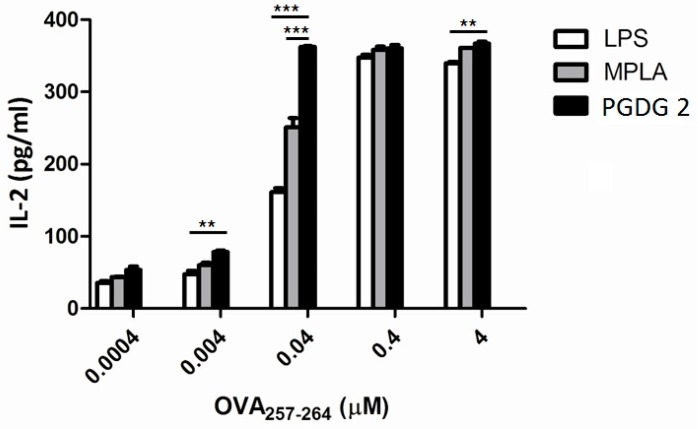
IL-2 release of mouse OTI hybridoma cell line B3Z in response to OVA_257–264_ SIINFEKL peptide. Bone marrow-derived dendritic cells (BM-DCs) were incubated with graded doses of synthetic OVA_257–264_ peptide in combination with 1 µg/mL LPS, MPLA or **2** and co-cultured with B3Z hybridoma cells for 40 h. Supernatants were assayed in duplicate. Mean ± SEM is reported, one representative experiment of two is shown. *p* < 0.001 by two-way ANOVA and the Bonferroni multiple comparison test. ** *p* < 0.01, *** *p* < 0.001.

We found that synthetic PGDG **2** at 1 µg/mL significantly improves DCs capability of activating an antigen-specific T cell response, especially when low doses (0.004 and 0.04 µM) of OVA_257–264_ peptide were used. In particular, at 0.04 µM OVA_257–264_ concentration, the IL-2 release by B3Z cells stimulated with PGDG-matured DCs yet reached a plateau with respect to LPS or MPLA matured DCs (Figure 8).

Microalgal PGDG emerged as a novel class of TLR-4 agonists. Since structurally related bacterial phosphatidylglycosylglycerides with phosphate at carbon 6′ of the sugar have never been tested for their immunomodulant potential or, more in general, for any biological activity [6,12,22], this represents the first report on immunostimulant activity of bioactive phosphatidylmonoglycosyldiacylglycerol lipids. Based on the chemical structure and similarity with LPS and LTA it is hard to find any structure/activity relationship suggesting that many chemical features can modulate ligand-receptor interaction. One of these is represented by fatty acyl composition that is likely responsible of the more potent effect of synthetic (C16:0/C18:0) PGDG **2** versus the natural pool of PGDG **1** rich in C20/C16 unsaturated fatty acids [50].

## 3. Materials and Methods 

### 3.1. General 

NMR spectra were recorded in CDCl_3_:CD_3_OD (1:1) on a 600 MHz Bruker Avance III spectrometer (Bruker BioSpin GmbH, Rheinstetten, Germany) equipped with a CryoProbeTM at 600.15 MHz. Chemical shifts values are reported in ppm (δ) and referenced to internal signals of residual protons (CD_3_OD ^1^H δ 3.34, ^13^C 49.0 ppm). High resolution mass spectra were acquired on a Q-Exactive Hybrid Quadrupole-Orbitrap Mass Spectrometer (Thermo Scientific, Milan, Italy) on-line with a UHPLC apparatus Infinity 1290 (Agilent Technologies, Santa Clara, CA, USA). HPLC analyses were performed on a Jasco system (PU-2089 Plus-Quaternary Gradient Pump equipped with a Jasco MD-2018 Plus Photodiode Array Detector (Jasco, Cremella, Italy). Solid Phase Extraction (SPE) was carried out using polystyrene-divinyl benzene resin (CHROMABOND® HR-X, Macherey-Nagel, Düren, Germany). GCMS analysis was performed by an ion-trap MS instrument in EI mode (70eV) (Thermo, Polaris Q) connected with a GC system (Thermo, GCQ) by a 5% phenyl/methyl polysiloxane column (30 m × 0.25 mm × 0.25 µm, Agilent, VF-5ms) using Helium as gas carrier. Sugar analysis was achieved on a HPAE-PAD (Dionex-Thermo Fisher Scientific, Milan, Italy). Chemicals were of analytical reagent grade and solvents of HPLC grade (Sigma-Aldrich, Milan, Italy) and were used without any further purifications. The synthetic peptide OVA_257–264_ (SIINFEKL) was purchased from Primm (Naples, Italy). Lipopolysaccharides from *E. coli* (serotype 055:B5) and MPLA were from Sigma-Aldrich.

### 3.2. Culture Condition

*Thalassiosira weissflogii* (Grun.) Fryxell et Hasle (CCMP 1336) was purchased from National Center for Marine Algae and Microbiota (Bigelow Laboratory for Ocean Sciences, East Boothbay, ME, USA).

The axenic strain was grown in a 10 L polycarbonate carboys, in f/2 culture medium, at room temperature (22.0 ± 1.0 °C) and gently bubbled with sterile air. The initial cell density was about 10,000 cells mL^−1^, and the inoculum was taken from 1 L of healthy exponentially growing culture. Artificial light intensity (200 µmol m^−2^ s^−1^) was provided by daylight fluorescent tubes with a 14 h:10 h light: dark photoperiod.

Cell growth was estimated by daily using a Burker counting chamber 0,1 mm depth (Merck, Leuven, Belgium;) under an inverted microscope AxioVision SE64 (Carl Zeiss, Thornwood, NY, USA).

Microalgae were harvested in the late stationary/beginning senescent phase by centrifugation in a swing out Allegra 12XR (Beckman Coulter Inc., Palo Alto, CA, USA) at 2300 g for 10 min, and stored at −80 °C until analyses.

### 3.3. Extraction, Fractionation and Identification of PGDG (**1**) 

The cell pellet of *T. weissflogii* (10.2 g) obtained as above described was extracted with methanol (3 × 50 mL), sonicated and centrifuged to remove cell debris. The methanol phase was filtered through paper and concentrated under vacuum without heating by rotavapor (R-300, Buchi Italia, Cornaredo, Italy). The raw extract (550 mg) was fractionated on LH-20 resin eluted with CHCl_3_:CH_3_OH 1:1 affording 13 samples A-O. Fraction B (15 mg) was further purified by HPLC on a Luna Phenyl-hexyl column (Phenomenex, 250 × 10 mm, 5 µm, 3 mL/min), by CH_3_OH/H_2_O elution gradient from 80% to 100% CH_3_OH in 10 min, holding at 100% for 10 min. PGDG **1** (2 mg) was isolated as an unresolved pool of species eluting in the range Tr = 14–15.8 min. 

PGDG (**1**). Yellowish amorphous solid. NMR data see Table 1. HR-ESI MS data see Supporting Information Figure S19 and Table S1; Table S2 for Fatty acid analysis. 

### 3.4. Fatty Acid Analysis

200 µg of PGDG pool (**1**) were dissolved in MeOH (0.5 mL), treated with Na_2_CO_3_ and heated to 53 °C for 20 h. The reaction mixture was diluted with a solution of HCl 0.2 M and extracted with Et_2_O (3 × 2 mL); the organic solvent was evaporated under reduced pressure and then analyzed in GCMS by using the following gradient: Initial 160 °C holding for 3 min; then 5 °C/min up to 260 °C followed by 30 °C/min up to 310 °C, holding for 3 min at 310 °C. Inlet temp 260 °C; Split flow 10 mL/min. Full scan *m*/*z* 50–450. Results are reported in Supporting Information, Table S1.

### 3.5. LC-ESI^-^/MS/MS Analysis

LC-MS/MS analysis was carried out according to [19]. Briefly, 200 µg of PGDG pool (**1**) were dissolved in MeOH (1 mL) and analysed in negative ionization mode in the mass range 1000–1800 *m*/*z* on a Q-Exactive mass spectrometer coupled to a UHPLC system Infinity 1290 equipped with a Kinetex Biphenyl 2.6 µm, 150 × 2.1 mm column, (Phenomenex, Castel Maggiore, Bologna, Italy). Eluent A: water and eluent B: MeOH. The elution program consisted of a gradient from 40% to 80% B in 2 min, then to 100% B in 13 min, holding at 100% B for 7 min. Flow rate was 0.3 mL/min. The injection volume was 10 μL, the autosampler was maintained at 10°C and the column at 28 °C. Results are reported in Supporting Information, Table S2.

### 3.6. Monosaccharide Analysis

The aqueous phase from previous extraction was transferred in a reaction vial and subjected to hydrolysis with TFA 6 M, at 110 °C for 4 h. The reaction mixture was evaporated under nitrogen stream, redissolved with distilled water and directly analysed by High Pressure Anion Exchange column (Carbopac PA1) connected with a Pulsed Amperometric Detector (HPAE-PAD, eluting with 18 mM NaOH, at 0.25 mL/min. Sugar identification was achieved by co-injection with commercial standards.

### 3.7. Synthetic Procedure for PGDG **2**

6′-*O*-trityl-d-1′,2′,3′,4′-Tetracetyl-galactopyranose (**I**): d-galactose (0.06 g, 0.334 mmol) was dissolved in pyridine (0.6 mL); 1.3 equivalents of trityl chloride (0.121 g, 0.434 mmol) was added slowly in different portions; the reaction mixture was stirred for 2 h at 90 °C; subsequently, acetic anhydride (0.3 mL) was added and after stirring overnight the mixture was evaporated under a stream of nitrogen and purified by silica gel chromatography using a light petroleum ether/diethyl ether gradient to give 6′-*O*-trityl-d-1′,2′,3′,4′-tetracetyl-galactopyranose (**I**) (0.165 g, 0.28 mmol, 84%); R_f_ (light petroleum ether/diethyl ether 3:7) = 0.49; ^1^H-NMR (400 MHz, CDCl_3_): (mix of α- and β-anomers) δ 7.52-7.12 (overlapped, aromatic protons, 15H), 6.39 (bs, 1H), 5.71 (d, *J* = 8.2 Hz, 1H), 5.62 (bs, 1H), 5.53 (m, 1H), 5.39 (m, 1H), 5.28 (m, 1H), 5.09 (m, 1H), 4.32 (m, 1H), 3.39 (m, 1H), 3.18 (m, 1H) 2.31-1.98 (overlapped, acetate methyls); HRESIMS *m*/*z* 613.2073 [M + Na]^+^ (calcd for C_33_H_34_O_10_Na^+^, 613.2044).

d-1′,2′,3′,4′-Tetracetyl-galactopyranose (**II**): 6′-*O*-trityl-d-1′,2′,3′,4′-tetracetyl-galactopyranose (**I**) (0.165 g, 0.28 mmol) was dissolved in acetic acid (0.64 mL) at 10 °C and bromidric acid (33% in acetic acid, 0.056 mL) was added; after one minute of stirring the mixture was filtered and washed with water (0.4 mL); the filtrate was diluted with 2 mL of water and extracted three times with dichloromethane (1.3 mL). The organic phase was purified by silica gel chromatography using a light petroleum ether/diethyl ether gradient to give d-1′,2′,3′,4′-tetracetyl-galactopyranose (II) (0.077 g, 0.22 mmol 79%); R_f_ (light petroleum ether/diethyl ether 3:7) = 0.20; ^1^H-NMR (400 MHz, CDCl_3_) (mix of α- and β-anomers): δ 6.31 (bs, 1H), 5.68 (d, *J* = 7.7 Hz, 1H), 5.41 (bd, *J* = 2.9 Hz, 1H), 5.31 (m, 1H), 5.12 (m, 1H), 3.69 (m, 1H), 3.49 (m, 1H), 2.16-1.91 (overlapped, acetate methyls); HRESIMS *m*/*z* 371.0957 [M + Na]^+^ (calcd for C_14_H_20_O_10_Na^+^, 371.0949).

6′-*O*-dimethylphosphoryl-d-1′,2′,3′,4′-Tetracetyl-galactopyranose (**III**): d-1′,2′,3′,4′-tetracetyl-galactopyranose (**II**) (0.077 g, 0.22 mmol) was dissolved in pyridine (0.28 mL); trimethylphosphite (0.033 mL, 0.288 mmol) and carbon tetrabromide (0.084 g, 0.266 mmol) were added at 0°C. The mixture was stirred in argon atmosphere for 2 h at room temperature. The solvent was evaporated and the residue was purified by by silica gel chromatography using a chloroform/methanol gradient to give 6′-*O*-dimethylphosphoryl-d-1′,2′,3′,4′-tetracetyl-galactopyranose (**III**) (0.059 g, 0.128 mmol 58%); R_f_ (diethyl ether) = 0.15; ^1^H-NMR (400 MHz, CDCl_3_) (mix of α- and β-anomers): δ 6.34 (bs, 1H), 5.68 (d, *J* = 7.8 Hz, 1H), 5.4 (bd, *J* = 2.7 Hz, 1H), 5.29 (m, 1H), 5.08 (m, 1H), 4.15 (m, 1H), 4.10-4.02 (m, 2H), 3.86-3.70 (overlapped, phosphate O-methyls), 2.19-1.95 (overlapped, acetate methyls); HRESIMS *m*/*z* 479.0926 [M + Na]^+^ (calcd for C_16_H_25_O_13_PNa^+^, 479.0925).

6′-*O*-dimethylphosphoryl-d-2′,3′,4′-triacetyl-galactopyranose (**IV**): 6′-*O*-dimethylphosphoryl-d-1′,2′,3′,4′-tetracetyl-galactopyranose (**III**) (0.059 g, 0.128 mmol) was dissolved in tetrahydrofurane (0.36 mL); benzylamine (0.0176 mL, 0.192 mmol) was added; the mixture was stirred overnight at room temperature and after evaporation of solvent was purified by silica gel chromatography using a chloroform/methanol gradient to give 6′-*O*-dimethylphosphoryl-d-2′,3′,4′-triacetyl-galactopyranose (**IV**) (0.0356 g, 0.086 mmol 67%); R_f_ (chloroform:methanol 95:5) = 0.49; ^1^H-NMR (400 MHz, CDCl_3_) (mix of α- and β-anomers): δ 5.49 (bd, *J* = 3.0 Hz, 1H), 5.43 (bs, 1H), 5.13 (d, *J* = 7.7 Hz, 1H), 5.29 (m, 1H), 5.08 (m, 1H), 4.95 (m, 1H), 4.51 (m, 1H), 4.12-4.01 (m, 2H), 3.88–3.69 (overlapped, phosphate *O*-methyls), 2.20-1.95 (overlapped, acetate methyls); HRESIMS *m*/*z* 437.0832 [M + Na]^+^ (calcd for C_14_H_23_O_12_PNa^+^, 437.0819).

6′-*O*-dimethylphosphoryl-1′-α-d-2′,3′,4′-triacetyl-galactopyranosyl-trichloroacetimidate (**V**): 6′-*O*-dimethylphosphoryl-d-2′,3′,4′-triacetyl-galactopyranose (**IV**) (0.0356 g, 0.086 mmol) was dissolved in dichloromethane (0.38 mL); 1,8-Diazabicyclo[5.4.0]undec-7-ene (DBU) (0.00266 mL, 0.0172 mmol) and trichloroacetonitrile (0.087 mL, 0.86 mmol) were added and the mixture was stirred on molecular sieves for 2 h at 0 °C; after evaporation of solvent the mixture was purified by silica gel chromatography using a dichloromethane/methanol gradient to give 6′-*O*-dimethylphosphoryl-1′-β-d-2′,3′,4′-triacetyl-galactopyranosyl-trichloroacetimidate (**V**) (0.0412 g, 0.074 mmol 86%); R_f_ (chloroform:methanol 95:5) = 0.70; ^1^H-NMR (400 MHz, CDCl_3_): δ 8.65 (s, NH), 6.59 (bd, *J* = 3.7 Hz, 1H), 5.58 (bd, *J* = 2.3 Hz, 1H), 5.40 (dd, *J* = 2.4, 10.6 Hz, 1H), 5.33 (dd, *J* = 3.7, 10.6 Hz, 1H), 4.42 (m, 1H), 4.12–3.96 (m, 2H), 3.77-3.68 (overlapped, phosphate *O*-methyls, 6H), 2.21–1.96 (acetate methyls, 9H); HRESIMS *m*/*z* 579.9928 [M + Na]^+^ (calcd for C_16_H_23_Cl_3_NO_12_PNa^+^, 579.9916).

6′-*O*-dimethylphosphoryl-1′-β-d-2′,3′,4′-triacetyl-galactopyranosyl-*sn*-isopropylidene-glycerol (**VI**): 6′-*O*-dimethylphosphoryl-1′-β-d-2′,3′,4′-triacetyl-galactopyranosyl-trichloroacetimidate (**V**) (0.0412 g, 0.074 mmol) was dissolved in anhydrous dichloromethane (0.44 mL) and 1.5 equivalents of 1,2-(*S*)-*O*-isopropylidene glycerol was added; the reaction mixture was kept at −20 °C on activated 3 Å molecular sieves under argon; boron trifluoride etherate (0.00254 mL, 0.0103 mmol) was added and stirring was maintained for 2 h; after another addition of boron trifluoride etherate (0.00254 mL, 0.0103 mmol), the temperature was raised to −10 °C and the reaction mixture was stirred overnight at that temperature. After neutralization with triethylamine (4 μL) and filtration on celite, the mixture was evaporated under reduced pressure and purified by silica gel chromatography using a dichloromethane/methanol gradient to give 6′-*O*-dimethylphosphoryl-1′-β-d-2′,3′,4′-triacetyl-galactopyranosyl-*sn*-isopropylidene-glycerol (**VI**) (0.0258 g, 0.048 mmol, 66%); R_f_ (chloroform:methanol 95:5) = 0.65; ^1^H-NMR (400 MHz, CDCl_3_): δ 5.38 (bd, *J* = 3.3 Hz, 1H), 5.14 (dd, *J* = 8.04, 10.5 Hz, 1H), 4.97 (dd, *J* = 3.3, 10.5 Hz, 1H), 4.55 (d, *J* = 8.0 Hz, 1H), 4.34 (m, 1H), 4.14–3.78 (m, 7H), 3.75–3.68 (overlapped, phosphate *O*-methyls, 6H), 2.11 (s, acetate methyl, 3H), 2.02 (s, acetate methyl, 3H), 1.93 (s, acetate methyl, 3H), 1.36 (s, acetonide methyl, 3H), 1.29 (s, acetonide methyl, 3H); HRESIMS *m*/*z* 551.1512 [M + Na]^+^ (calcd for C_20_H_33_O_14_PNa^+^, 551.1500).

6′-*O*-dimethylphosphoryl-1′-β-d-2′,3′,4′-triacetyl-galactopyranosyl-*sn*-glycerol (**VII**): 6′-*O*-dimethylphosphoryl-1′-β-d-2′,3′,4′-triacetyl-galactopyranosyl-*sn*-isopropylidene-glycerol (**VI**) (0.0258 g, 0.048 mmol) was dissolved in methanol-water solution (0.66 mL, 9/1) and Dowex 50WX8-H^+^ resin (0.4 g) was added; after 3 h under stirring, the reaction mixture was filtered, evaporated and purified by silica gel chromatography using a gradient of dichloromethane/methanol to give 6′-*O*-dimethylphosphoryl-1′-β-d-2′,3′,4′-triacetyl-galactopyranosyl-*sn*-glycerol (VII) (0.0138 g, 0.028 mmol, 59%); R_f_ (chloroform:methanol 95:5) = 0.30; ^1^H-NMR (400 MHz, CDCl_3_): δ 5.43 (bd, *J* = 3.1 Hz, 1H), 5.20 (dd, *J* = 7.4, 10.1 Hz, 1H), 5.03 (dd, *J* = 3.2, 10.1 Hz, 1H), 4.53 (d, *J* = 7.4 Hz, 1H), 4.17–4.10 (m, 1H), 4.08–4.01 (m, 1H), 3.97 (m, 1H), 3.88–3.84 (m, 2H), 3.79–3.75 (overlapped, phosphate methyls, 6H), 3.70 (m, 2H), 3.59 (dd, *J* = 4.8, 11.4 Hz, 1H), 2.16 (s, acetate methyl, 3H), 2.07 (s, acetate methyl, 3H), 1.98 (s, acetate methyl, 3H); HRESIMS *m*/*z* 511.1197 [M + Na]^+^ (calcd for C_17_H_29_O_14_PNa^+^, 511.1187).

6′-*O*-dimethyl-phosphoryl-1′-β-d-2′,3′,4′-triacetyl-galactopyranosyl-1,2-distearoyl-*sn*-glycerol (**VIII**): 6′-*O*-dimethylphosphoryl-1′-β-d-2′,3′,4′-triacetyl-galactopyranosyl-*sn*-glycerol (**VII**) (0.0138 g, 0.028 mmol) was dissolved in anhydrous dichloromethane (0.4 mL); stearic acid (0.032 g, 0.112 mmol), dicyclohexylcarbodiimide (0.0232 g, 0.112 mmol) and DMAP (0.0069 g, 0.028 mmol) were added under argon; the reaction mixture was stirred overnight at room temperature; after evaporation under reduced pressure, the mixture was purified by silica gel chromatography using a gradient of dichloromethane/methanol to give 6′-*O*-dimethyl-phosphoryl-1′-β-d-2′,3′,4′-triacetyl-galactopyranosyl-1,2-distearoyl-*sn*-glycerol (**VIII**) (0.0264 g, 0.026 mmol, 94%); R_f_ (chloroform:methanol 95:5) = 0.80; ^1^H-NMR (400 MHz, CDCl_3_): δ 5.44 (bd, *J* = 2.6 Hz, 1H), 5.19–5.14 (overlapped, 2H), 5.02 (dd, *J* = 3.8, 10.5 Hz, 1H), 4.48 (d, *J* = 7.3 Hz, 1H), 4.13 (dd, *J* = 2.7, 11.7 Hz, 1H), 4.16–4.07 (m, 3H), 3.95 (m, 1H), 3.75 (d, *J* = 11.4 Hz, phosphate methyls, 6H), 3.65 (dd, *J* = 5.7, 11.7Hz, 1H), 2.30 (t, *J* = 7.1 Hz, α-methylenes of acyl portions, 4H), 2.16 (s, acetate methyl, 3H), 2.06 (s, acetate methyl, 3H), 1.98 (s, acetate methyl, 3H), 1.61 (overlapped, β-methylenes of acyl portions, 4H), 1.37–1.20 (m, aliphatic protons), 0.88 (t, *J* = 7.0 Hz, methyls, 6H); HRESIMS *m*/*z* 1043.6418 [M + Na]^+^ (calcd for C_53_H_97_O_16_PNa^+^, 1043.6406).

6′-*O*-phosphoryl-1′-β-d-2′,3′,4′-triacetyl-galactopyranosyl-1,2-distearoyl-*sn*-glycerol (**IX**): 6′-*O*-dimethyl-phosphoryl-1′-β-d-2′,3′,4′-triacetyl-galactopyranosyl-1,2-distearoyl-*sn*-glycerol (**VIII**) (0.0264 g, 0.026 mmol) was dissolved in acetonitrile (1.36 mL); TMS-Cl (0.008 mL, 0.062 mmol) and sodium iodide (0.0097 g, 0.068 mmol) were added and after stirring in the dark at room temperature overnight, the mixture was filtered and washed with dichlorometane; the filtrate was evaporated to get 6′-*O*-phosphoryl-1′-β-d-2′,3′,4′-triacetyl-galactopyranosyl-1,2-distearoyl-*sn*-glycerol (IX) (0.022 g, 0.022 mmol, 85%); R_f_ (chloroform:methanol 85:15) = 0.10; ^1^H-NMR (400 MHz, CDCl_3_): δ 5.44 (bs, 1H), 5.22–5.15 (overlapped, 2H), 5.02 (bd, *J* = 10.3 Hz, 1H), 4.50 (d, *J* = 7.1 Hz, 1H), 4.13–3.92 (overlapped, 5H), 3.68 (m, 1H), 2.30 (t, *J* = 7.1 Hz, α-methylenes of acyl portions, 4H), 2.16 (s, 3H, acetate methyl), 2.05 (s, 3H, acetate methyl), 1.98 (s, 3H, acetate methyl), 1.66–1.54 (overlapped, β-methylenes of acyl portions, 4H), 1.36–1.18 (m, aliphatic protons), 0.88 (t, *J* = 6.8 Hz, methyls, 6H); HRESIMS *m*/*z* 991.6127 [M − H]^−^ (calcd for C_51_H_92_O_16_P^−^, 991.6129).

3-*O*-[6′-*O*-(3″-phosphoryl-*sn*-isopropylidene-glycerol)-1′-β-d-2′,3′,4′-triacetyl-galactopyranosyl]-1,2-distearoyl-*sn*-glycerol (**X**): 6′-*O*-phosphoryl-1′-β-d-2′,3′,4′-triacetyl-galactopyranosyl]-1,2-distearoyl-*sn*-glycerol (**IX**) (0.022 g, 0.022 mmol) and 1,2-(S)-*O*-isopropylidene glycerol (0.0058 g, 0.044 mmol) were dissolved in small quantities of pyridine and evaporated different times by nitrogen flow and finally were dissolved in anhydrous pyridine (0.3 mL) and trichloroacetonitrile (0.0226 mL, 0.22 mmol) was added; after stirring at 75 °C under argon atmosphere overnight, the mixture was evaporated and purified by silica gel chromatography using a gradient of dichloromethane/methanol to give 3-*O*-[6′-*O*-(3″-phosphoryl-*sn*-isopropylidene-glycerol)-1′-β-d-2′,3′,4′-triacetyl-galactopyranosyl]-1,2-distearoyl-*sn*-glycerol (**X**) (0.0158 g, 0.0143 mmol, 65%); R_f_ (chloroform:methanol 85:15) = 0.25; ^1^H-NMR (400 MHz, MeOD): δ 5.49 (bs, 1H), 5.24 (m, 1H), 5.16-5.09 (overlapped, 2H), 4.70 (d, *J* = 7.0 Hz, 1H), 4.38-4.23 (overlapped, 2H), 4.15 (dd, *J* = 7.0, 12.2 Hz, 1H), 4.15–4.10 (overlapped, 2H), 4.02 (m, 1H), 3.96–3.89 (overlapped, 3H), 3.84 (m, 1H), 3.75 (m, 1H), 2.39–2.31 (overlapped, α-methylenes of acyl portions, 4H), 2.19 (s, 3H, acetate methyl), 2.09 (s, 3H, acetate methyl), 1.99 (s, 3H, acetate methyl), 1.68–1.60 (overlapped, β-methylenes of acyl portions, 4H), 1.43 (s, methyl of acetonide, 3H), 1.37 (s, methyl of acetonide, 3H), 1.34–1.15 (m, aliphatic protons), 0.93 (t, *J* = 6.9 Hz, methyls, 6H); HRESIMS *m*/*z* 1105.6798 [M−H]^−^ (calcd for C_57_H_102_O_18_P^−^, 1105.6809).

3-*O*-[6′-*O*-(3″-methylphosphoryl-*sn*-isopropylidene-glycerol)-1′-β-d-2′,3′,4′-triacetyl-galactopyranosyl]-1,2-distearoyl-*sn*-glycerol (**XI**): 3-*O*-[6′-O-(3″-phosphoryl-*sn*-isopropylidene-glycerol)-1′-β-d-2′,3′,4′-triacetyl-galactopyranosyl]-1,2-distearoyl-*sn*-glycerol (**X**) (0.0158 g, 0.0143 mmol) was dissolved in anhydrous acetonitrile (0.54 mL); silver (I) oxide (0.00692 g, 0.03 mmol) and iodomethane (0.0286 mL, 0.46 mmol) were added; after stirring at 40°C overnight, the mixture was evaporated and purified by silica gel chromatography using a gradient of dichloromethane/methanol to give 3-*O*-[6′-*O*-(3″-methylphosphoryl-sn-isopropylidene-glycerol)-1′-β-d-2′,3′,4′-triacetyl-galactopyranosyl]-1,2-distearoyl-sn-glycerol (**XI**) (0.0085 g, 0.00758 mmol, 53%); R_f_ (chloroform:methanol 85:15) = 0.85; ^1^H-NMR (400 MHz, CDCl_3_): δ 5.43 (bd, *J* = 2.2 Hz, 1H), 5.22-5.16 (overlapped, 2H), 5.01 (m, 1H), 4.48 (d, *J* = 7.5 Hz, 1H), 4.35-4.28 (overlapped, 2H), 4.16–4.02 (overlapped, 4H), 3.99–3.92 (overlapped, 2H), 3.82-3.72 (overlapped, 7H), 2.30 (t, *J* = 7.1 Hz, α-methylenes of acyl portions, 4H), 2.16 (s, 3H, acetate methyl), 2.06 (s, 3H, acetate methyl), 1.98 (s, 3H, acetate methyl), 1.64–1.56 (overlapped, β-methylenes of acyl portions, 4H), 1.43 (s, methyl of acetonide, 3H), 1.36 (s, methyl of acetonide, 3H), 1.33–1.21 (m, aliphatic protons), 0.88 (t, *J* = 7.0 Hz, methyls, 6H); HRESIMS *m*/*z* 1143.6910 [M + Na]^+^ (calcd for C_58_H_105_O_18_PNa^+^, 1143.6931).

3-*O*-[6′-*O*-(3″-methylphosphoryl-*sn*-glycerol)-1′-β-d-2′,3′,4′-triacetyl-galactopyranosyl]-1,2-distearoyl-*sn*-glycerol (**XII**): 3-*O*-[6′-*O*-(3″-methylphosphoryl-*sn*-isopropylidene-glycerol)-1′-β-d-2′,3′,4′-triacetyl-galactopyranosyl]-1,2-distearoyl-*sn*-glycerol (**XI**) (0.0085 g, 0.00758 mmol) was dissolved in methanol-water-chloroform suspension (0.72 mL, 97/3/30) and Dowex 50WX8-H^+^ resin (0.17 g) was added; after 7 h under stirring at 60 °C, the reaction mixture was filtered, evaporated and purified by silica gel chromatography using a gradient of dichloromethane/methanol to give 3-*O*-[6′-*O*-(3″-methylphosphoryl-*sn*-glycerol)-1′-β-d-2′,3′,4′-triacetyl-galactopyranosyl]-1,2-distearoyl-*sn*-glycerol (**XII**) (0.0052 g, 0.005 mmol, 66%); R_f_ (chloroform:methanol 95:5) = 0.45; ^1^H-NMR (400 MHz, CDCl_3_): δ 5.45 (bs, 1H), 5.24–5.14 (overlapped, 2H), 5.02 (m, 1H), 4.50 (d, *J* = 7.9 Hz, 1H), 4.31 (dd, *J* = 3.0, 11.9 Hz), 4.17-4.09 (overlapped, 3H), 4.06 (m, 1H), 3.99–3.91 (overlapped, 2H), 3.84–3.74 (overlapped, 6H), 3.71–3.63 (overlapped, 3H), 2.30 (t, *J* = 7.2 Hz, α-methylenes of acyl portions, 4H), 2.18 (s, 3H, acetate methyl), 2.06 (s, 3H, acetate methyl), 1.99 (s, 3H, acetate methyl), 1.68–1.53 (overlapped, β-methylenes of acyl portions, 4H), 1.36–1.27 (m, aliphatic protons), 0.88 (t, *J* = 6.8 Hz, methyls, 6H); HRESIMS *m*/*z* 1103.6619 [M + Na]^+^ (calcd for C_55_H_101_O_18_PNa^+^, 1103.6618).

3-*O*-[6′-*O*-(1″,2″-dipalmitoyl-3″-methylphosphoryl-*sn*-glycerol)-1′-β-d-2′,3′,4′-triacetyl-galactopyranosyl]-1,2-distearoyl-*sn*-glycerol (**XIII**): 3-*O*-[6′-*O*-(3″-methylphosphoryl-*sn*-glycerol)-1′-β-d-2′,3′,4′-triacetyl-galactopyranosyl]-1,2-distearoyl-*sn*-glycerol (**XII**) (0.0052 g, 0.005 mmol) was dissolved in anhydrous dichloromethane (0.3 mL); palmitic acid (0.00532 g, 0.022 mmol), dicyclohexylcarbodiimide (0.0051 g, 0.022 mmol) and DMAP (0.00194 g, 0.0152 mmol) were added; the reaction mixture was stirred overnight at 45 °C; after evaporation of solvent under reduced pressure, the mixture was purified by silica gel chromatography using a gradient of chloroform/methanol to give 3-*O*-[6′-*O*-(1″,2″-dipalmitoyl-3″-methylphosphoryl-*sn*-glycerol)-1′-β-d-2′,3′,4′-triacetyl-galactopyranosyl]-1,2-distearoyl-*sn*-glycerol (**XIII**) (0.0078 g, 0.005 mmol, 66%); R_f_ (chloroform:methanol 95:5) = 0.90; ^1^H-NMR (400 MHz, CDCl_3_): δ 5.43 (bs, 1H), 5.25–5.14 (overlapped, 2H), 5.02 (dd, *J* = 2.7, 10.8 Hz, 1H), 4.49 (d, *J* = 7.4 Hz, 1H), 4.36–4.25 (overlapped, 3H), 4.20–4.08 (overlapped, 4H), 4.04 (m, 1H), 3.98–3.93 (overlapped, 2H), 3.75 (d, *J* = 11.1 Hz, phosphate methyl, 3H), 3.65 (dd, *J* = 6.1, 10.6 Hz, 1H), 2.36–2.25 (overlapped, α-methylenes of acyl portions, 8H), 2.16 (s, 3H, acetate methyl), 2.06 (s, 3H, acetate methyl), 1.98 (s, 3H, acetate methyl), 1.64–1.55 (overlapped, β-methylenes of acyl portions, 8H), 1.34–1.17 (m, aliphatic protons), 0.88 (t, *J* = 6.9 Hz, methyls, 12H); HRESIMS *m*/*z* 1580.1205 [M+Na]^+^ (calcd for C_87_H_161_O_20_PNa^+^, 1580.1211).

3-*O*-[6′-*O*-(1″,2″-dipalmitoyl-3″-phosphoryl-*sn*-glycerol)-1′-β-d-2′,3′,4′-triacetyl-galactopyranosyl]-1,2-distearoyl-*sn*-glycerol (**XIV**): 3-*O*-[6′-*O*-(1″,2″-dipalmitoyl-3″-methylphosphoryl-*sn*-glycerol)-1′-β-d-2′,3′,4′-triacetyl-galactopyranosyl]-1,2-distearoyl-*sn*-glycerol (XIII) (0.0078 g, 0.005 mmol) was dissolved in acetonitrile (0.4 mL); TMS-Cl (0.0015 mL, 0.01 mmol) and sodium iodide (0.0014 g, 0.01 mmol) were added and after stirring in the dark at room temperature overnight, the mixture was filtered and washed with dichlorometane; the filtrate was evaporated to get 3-*O*-[6′-*O*-(1″,2″-dipalmitoyl-3″-phosphoryl-*sn*-glycerol)-1′-β-d-2′,3′,4′-triacetyl-galactopyranosyl]-1,2-distearoyl-*sn*-glycerol (**XIV**) (0.00524 g, 0.0034 mmol, 68%); R_f_ (chloroform:methanol 95:5) = 0.30; ^1^H-NMR (400 MHz, CDCl_3_): δ 5.42 (bs, 1H), 5.24–5.11 (overlapped, 2H), 5.00 (dd, *J* = 2.6, 10.3 Hz, 1H), 4.53 (d, *J* = 7.0 Hz, 1H), 4.35-4.24 (overlapped, 3H), 4.15–4.24 (overlapped, 5H), 4.02–3.87 (overlapped, 3H), 3.68 (dd, *J* = 4.7, 10.9 Hz, 1H), 2.35–2.22 (overlapped, α-methylenes of acyl portions, 8H), 2.14 (s, 3H, acetate methyl), 2.04 (s, 3H, acetate methyl), 1.95 (s, 3H, acetate methyl), 1.63–1.51 (overlapped, β-methylenes of acyl portions, 8H), 1.32–1.15 (m, aliphatic protons), 0.86 (t, *J* = 6.5 Hz, methyls, 12H); HRESIMS *m*/*z* 1542.1059 [M−H]^−^ (calcd for C_86_H_158_O_20_P^−^, 1542.1090).

3-*O*-[6′-*O*-(1″,2″-dipalmitoyl-3″-phosphoryl-sn-glycerol)-1′-β-d-galactopyranosyl]-1,2-distearoyl-*sn*-glycerol (2): 3-*O*-[6′-*O*-(1″,2″-dipalmitoyl-3″-phosphoryl-*sn*-glycerol)-1′-β-d-2′,3′,4′-triacetyl-galactopyranosyl]-1,2-distearoyl-*sn*-glycerol (**XIV**) (0.00524 g, 0.0034 mmol) was dissolved in aq. ethanol (85%) (0.4 mL), hydrazine monohydrate (0.0025 g, 0.05 mmol) was added, and the reaction mixture was stirred for 7 h at 48°C. After evaporation of solvent under a stream of nitrogen, the residue was purified by silica gel chromatography using a gradient of chloroform/methanol to give 3-*O*-[6′-*O*-(1″,2″-dipalmitoyl-3″-phosphoryl-*sn*-glycerol)-1′-β-d-galactopyranosyl]-1,2-distearoyl-s*n*-glycerol (2) (2.1 mg, 0.0015 mmol, 44%); R_f_ (chloroform/methanol 8:2) = 0.40; See Table 1 and Supporting information for NMR data. HRESIMS *m*/*z* 1416.0792 (calcd for C_80_H_152_O_17_P^-^, 1416.0773).

### 3.8. Immunological Assays

#### 3.8.1. moDCs Generation

For each assay, human peripheral blood mononuclear cells (PBMC) were isolated from two healthy donors by routine Ficoll density gradient centrifugation. Monocytes were purified from human peripheral blood mononuclear cells using MACS CD14 microbeads (Miltenyi Biotech, Auburn, CA, USA) according to the manufacturer’s instructions. Purity was checked by staining with a FITC-conjugated anti-CD14 antibody (Milteny Biotech, Auburn, CA, USA) and FACS analysis. Immature DCs were obtained by incubating monocytes at 8 × 10^5^/mL in RPMI 1640 medium supplemented with 10% fetal calf serum (FCS), 1% l-glutamine 2 mM, 1% penicillin and streptomycin, human IL-4 (5 ng/mL) and human GM-CSF (100 ng/mL) for five days.

#### 3.8.2. Cells Staining and Stimulation

After five days in culture, surface staining was performed on monocyte-derived dendritic cells (MoDCs) for flow cytometry analysis with mouse anti-human HLA-DR FITC, CD83 PE, CD86 APC, CD54 PeVio770, CD14 FITC, CD3 PercP (all from Miltenyi Biotech) and analyzed by flow-cytometer LSRFortessa™ X-20 cell analyzer (BD Bioscience, Frankin Lake, NJ, USA) according to standard protocol. MoDC were then incubated with natural and synthetic compound in 12-well plates at concentration of 8 × 10^5^ cell/mL. Stimulation with PAM2CSK4 1 µg/mL (Invivogen, San Diego, CA, USA) was used as positive control. Cells treated only with vehicle (DMSO) were used as the control. For the experiment with the anti TLR-4 neutralizing antibody, cells were incubated 30 min with only the antibody (1 µg/mL) at 37 °C before addition of PGDG. After 24 h, expression of all surface markers was estimated again by staining with fluorochrome-conjugated antibodies.

#### 3.8.3. Real Time PCR Analysis 

Total RNA was isolated using Trizol Reagent, according to the manufacturer’s protocol. RNA quantity and purity were measured with a NanoDrop 2000 spectrophotometer (Thermo Scientific, Waltham, MA, USA). Sample purity was checked by A260/A280 ratios between 1.80 and 2.00. Extracted RNAs from all preparations were in this range. Cytokines mRNA expression was measured by quantitative Real Time-PCR. Results are expressed as means ± SD. 

#### 3.8.4. TLR-2 and TLR-4 Assays

Human TLR-4/NF-kB/SEAP and TLR-2/NF-kB/SEAP (SEAPorter™) HEK 293 reporter cells (Novus Biologicals, Littleton, CO, USA) were plated in 24-well plates at 2 × 10^5^ cells/well. After 16 h TLR4 cells for functional assays were transiently transfected with MD2/CD14 plasmid vector (Invivogen, San Diego, CA, USA) for 24 h. Cells were then stimulated with compounds for 24 h. LPS for TLR-4 cell line and PAM2CSK4 for TLR-2 cell line were used as positive control under the same conditions. SEAP (Secreted Alkaline Phosphatase) was analyzed using the SEAPorter Assay (Novus Biologicals, Littleton, CO, USA) in according to manufacturer’s instructions. Quantitative data (ng/mL) were obtained by a standard curve for the SEAP protein.

#### 3.8.5. BM-DCs Generation

Eight/nine-week-old female C57BL/6 mice were purchased from Charles River (Lecco, Italy) and housed in IGB “A. Buzzati-Traverso” Animal House Facility under standard pathogen-free conditions abiding institutional guidelines. Bone marrow-derived dendritic cells (BM-DCs) were produced from precursors isolated from the bone marrow of C57BL/6 mice by culturing them with 200 U/mL recombinant murine granulocyte/macrophage colony-stimulating factor (GM-CSF) (Peprotech, Rocky Hill, NJ, USA) in RPMI 1640 (Lonza, Basel, Switzerland) medium supplemented with 10% FCS, 100 U/mL penicillin, 100 µg/mL streptomycin, 1 mM sodium pyruvate and 50 µM 2-mercaptoethanol. Immature DCs were collected at day 7 of culture and were assayed for dendritic cell phenotype by staining with the monoclonal antibody anti-CD11c-PE-Cy7 (HL3, BD Biosciences).

#### 3.8.6. B3Z CD8+ T Cell Activation Assay

1 × 10^6^/mL immature BM-DCs were incubated for 3 h with different concentrations of OVA_257–264_ synthetic peptide (from 0.0004 to 4 µM) in presence of 1 µg/mL of LPS, MPLA, or PGDG **2**. As control, DCs incubated only with medium were used. After the incubation, cells were washed and co-cultured (50,000/well) with the OTI hybridoma cell line B3Z (50,000/well) for 40 h. The IL-2 released into cell co-culture supernatants was measured by ELISA. Supernatants (0.1 mL/well) were assayed in duplicate using mouse IL-2 ELISA MAX™ Standard (Biolegend, San Diego, CA, USA), according to the manufacturer’s instructions. B3Z OTI hybridoma cell line, recognizing the OVA_257–264_ SIINFEKL determinant, was growth in complete RPMI 1640 (10% FCS 100 U/mL penicillin, 100 μg/mL streptomycin 1% Glutamine, 1% NEM, 1% Sodium Pyruvate, 50 µM 2-Mercaptoethanol) and was a kind gift of Dr. Bénédicte Manoury (INEM, INSERM U1151-CNRS UMR 8253, Hôpital Necker, Paris, France). 

#### 3.8.7. Statistical Analysis

Comparative analysis was performed using the analysis of variance (ANOVA) followed by the Bonferroni post hoc comparisons or the Tukey test for multiple comparison test. A p-value less than 0.05 was considered as statistically significant. All analyses were performed using the GraphPad Prism 5.00 for Windows software (GraphPad Software, San Diego, CA, USA). 

## 4. Conclusions

A rare pool of lipids, namely phosphatidylmonogalactosyl diacylglycerols PGDG (**1**), was isolated and characterized for the first time from the marine diatom *T. weissflogii*. Phosphogalactolipid **1** is a tetra-acyl derivative featured by (poly)unsaturated fatty acid chains on a 3-*O*-[6′-(*sn*-glycero-3″-phosphoryl)-β-d-galactopyranosyl]-*sn*-glycerol. Its molecular core structure was defined by spectroscopic methods and confirmed by total synthesis of the analog **2**, performed according to a versatile and general approach that gives access to a series of regio- and stereo-pure phosphatidyl-β-glycosyl-diacylglycerol derivatives for biological and pharmacological development. PGDG lipids gave immunostimolatory activity on DCs in the range 1–10 µg/mL and TLR-based assays evidenced a TLR-4 agonistic activity: this represents the first report of an immunomodulant activity for these uncommon lipids. Functional studies with hybridoma T cells revealed an adjuvant potential of synthetic PGDG **2**, significantly higher than standard lipid adjuvants such as LPS and MPLA at 1 µg/mL in presence of OVA_257–264_ antigen. These results encourage further studies to define the specific intracellular signaling pathway mediated by TLR-4, to assess structure-activity relationship through preparation of molecular analogs and to explore the full pharmacological potential of this class of bioactive lipids. 

## Figures and Tables

**Table 1:** ^1^H and ^13^C NMR data of natural (**1**) and synthetic PGDG (**2**) (600 MHz, CDCl_3_:CD_3_OD, 1:1).

	1		2			1		2	
	^13^C, ppm	^1^H, δ, m, *J* (Hz)	^13^C, ppm	^1^H, δ, m, *J* (Hz)		^13^C, ppm	^1^H, δ, m, *J* (Hz)	^13^C, ppm	^1^H, δ, m, *J* (Hz)
Gal					Fatty acyl				
**1′**	104.5	4.23, d, 7.2	104.5	4.23, d, 7.1	**C=O**	174.1–174.4		174.2, 174.6	
**2′**	71.8	3.53, dd, 9.7, 7.2	71.9	3.53, dd, 9.8, 7.1	**α-CH_2_**	34.0–34.8	2.34, m	34.6, 34.8	2.33, m
**3′**	73.7	3.51, dd, 9.7, 3.3	73.7	3.51, dd, 9.8, 3.2	**β-CH_2_**	25.1–25.5	1.62–1.69, m	25.5	1.61, m
**4′**	68.3	3.99, m	68.0	3.99, brd, 3.2	**CH_2_ (chain)**	29.6–30.3	1.27–1.32, m	29.7–30.3	1.27, m
**5′**	74.3	3.63, dd, 8.4, 5.7	74.3	3.64, dd, 8.4, 5.8	**HC=CH**	128.4–130.6	5.36, m		
**6′a**	63.5	4.09, m	63.3	4.08, m	**=CH*CH_2_*CH_2_-**	27.1–27.7	2.02–2.11, m		
**6′b**		3.92, m		3.93, m					
**Gly_1_**					**=CH*CH_2_*CH=**	26.2	2.82–2.85, m		
**1a**	63.3	4.40, brd, 12.1	63.2	4.39, dd, 12.2. 2.9	**-*CH_2_*CH_3_**	23.2–23.3	1.31, m	23.2	1.30, m
**1b**		4.23, m		4.24, m					
**2**	71.0	5.25, m	71.1	5.25, m	**=CHCH_2_*CH_2_*CH_3_**	23.5	1.38, m		
**3a**	68.1	3.99, m	68.2	3.96, dd, 10.7, 5.5	**=CH*CH_2_*CH_3_**	20.9	2.07, m		
**3b**		3.68, dd, 10.5, 5.6		3.68, dd, 10.7, 5.3					
**Gly_2_**					**-(CH_2_)_n_*CH_3_***	14.0–14.4	0.88, t, 7.1	14.3	0.89, t, 7.1
**1″a**	63.4	4.44, dd, 12.1, 3.0	63.3	4.44, dd, 12.1, 3.2	**=CHCH_2_CH_2_*CH_3_***	14.0–14.4	0.92, t, 7.2		
**1″b**		4.20, m		4.21, m					
**2″**	71.2	5.23, m	71.0	5.25, m	**=CHCH_2_*CH_3_***	14.0–14.4	0.97, t, 7.5		
**3″**	64.1	4.00, m	64.3	3.99, m					

## Supplementary Materials

The following are available online at https://www.mdpi.com/1660-3397/17/2/103/s1, 1D (^1^H and ^13^C) and 2D NMR spectra of **1**. ^1^H and ^13^C NMR spectra of **2**, ^1^H NMR of synthetic intermediates **V-XIV**. Mass spectrometric (LCMS and GCMS) data of **1**. Immunostimulant assay on bioactive fraction from *T. weissflogii.*
