# Supplementary material for: Immunostimulatory Phosphatidylmonogalactosyldiacylglycerols (PGDG) from the Marine Diatom Thalassiosira weissflogii: Inspiration for a Novel Synthetic Toll-Like Receptor 4 Agonist
Source: Mar Drugs. 2019 Feb 9;17(2):103. doi: 10.3390/md17020103 (PMC6409857; doi:10.3390/md17020103)

Immunostimulatory phosphatidylmonogalactosyldiacylglycerols (PGDG) from the marine diatom *Thalassiosira weissflogii*: inspiration for a novel synthetic Toll-like receptor 4 agonist

Emiliano Manzo[1†], Carmela Gallo[1†], Rossella Sartorius[2], Genoveffa Nuzzo[1], Angela Sardo[1], Piergiuseppe De Berardinis[2], Angelo Fontana[1], Adele Cutignano[1*]

[1] CNR-Institute of Biomolecular Chemistry, via Campi Flegrei, 34 - 80078 Pozzuoli (Napoli) Italy
emanzo@icb.cnr.it; carmen.gallo@icb.cnr.it; nuzzo.genoveffa@icb.cnr.it; angela.sardo@icb.cnr.it; afontana@icb.cnr.it; acutignano@icb.cnr.it
[2] CNR- Institute of Protein Biochemistry, via Pietro Castellino, 111 - 80131 Napoli, Italy
r.sartorius@ibp.cnr.it; p.deberardinis@ibp.cnr.it
[*] Correspondence: acutignano@icb.cnr.it; Tel.: +39 081 8675313
[†]These authors have equally contributed

Supporting Information

1. NMR spectra
Figure S1. ^{1}H NMR spectrum of 1 (600MHz, $CDCl_3$/MeOD, 1:1).
Figure S2. ^{13}C NMR spectrum of 1 (600MHz, $CDCl_3$/MeOD, 1:1).
Figure S3. ^{1}H,^{1}H-COSY spectrum of 1 (600MHz, $CDCl_3$/MeOD, 1:1).
Figure S4. ^{1}H,^{1}H-TOCSY spectrum of 1 (600MHz, $CDCl_3$/MeOD, 1:1).
Figure S5. HSQC spectrum of 1 (600MHz, $CDCl_3$/MeOD, 1:1).
Figure S6. HMBC spectrum of 1 (600MHz, $CDCl_3$/MeOD, 1:1).
Figure S7. ^{1}H NMR spectrum of 2 (600MHz, $CDCl_3$/MeOD, 1:1).
Figure S8. ^{13}C NMR spectrum of 2 (600MHz, $CDCl_3$/MeOD, 1:1).
Figure S9. ^{1}H NMR spectrum of intermediate V (400MHz, $CDCl_3$).
Figure S10. ^{1}H NMR spectrum of intermediate VI (400MHz, $CDCl_3$).
Figure S11. ^{1}H NMR spectrum of intermediate VII (400MHz, $CDCl_3$).
Figure S12. ^{1}H NMR spectrum of intermediate VIII (400MHz, $CDCl_3$).
Figure S13. ^{1}H NMR spectrum of intermediate IX (400MHz, $CDCl_3$).
Figure S14. ^{1}H NMR spectrum of intermediate X (400MHz, MeOD).
Figure S15. ^{1}H NMR spectrum of intermediate XI (400MHz, $CDCl_3$).
Figure S16. ^{1}H NMR spectrum of intermediate XII (400MHz, $CDCl_3$)
Figure S17. ^{1}H NMR spectrum of intermediate XIII (400MHz, $CDCl_3$)
Figure S18. ^{1}H NMR spectrum of intermediate XIV (400MHz, $CDCl_3$)

2. MS data.
Figure S19. HR-ESI-MS spectrum of bioactive lipid pool isolated from the marine diatom *T. weissflogii*.
Table S1. Fatty acid composition (methyl derivatives) of 1 by GCMS analysis after methanolysis.
Table S2. Fatty acid composition of main species of the natural pool 1 by LC-ESI^{-}/MS/MS analysis.

3. Biological assays
Figure S20. Expression of cell surface markers in MoDCs by stimulation with LH-20 bioactive fraction from *T. weissflogii.*

Figure S1. ^{1}H NMR spectrum of 1 (600MHz, $CDCl_3$/MeOD, 1:1).

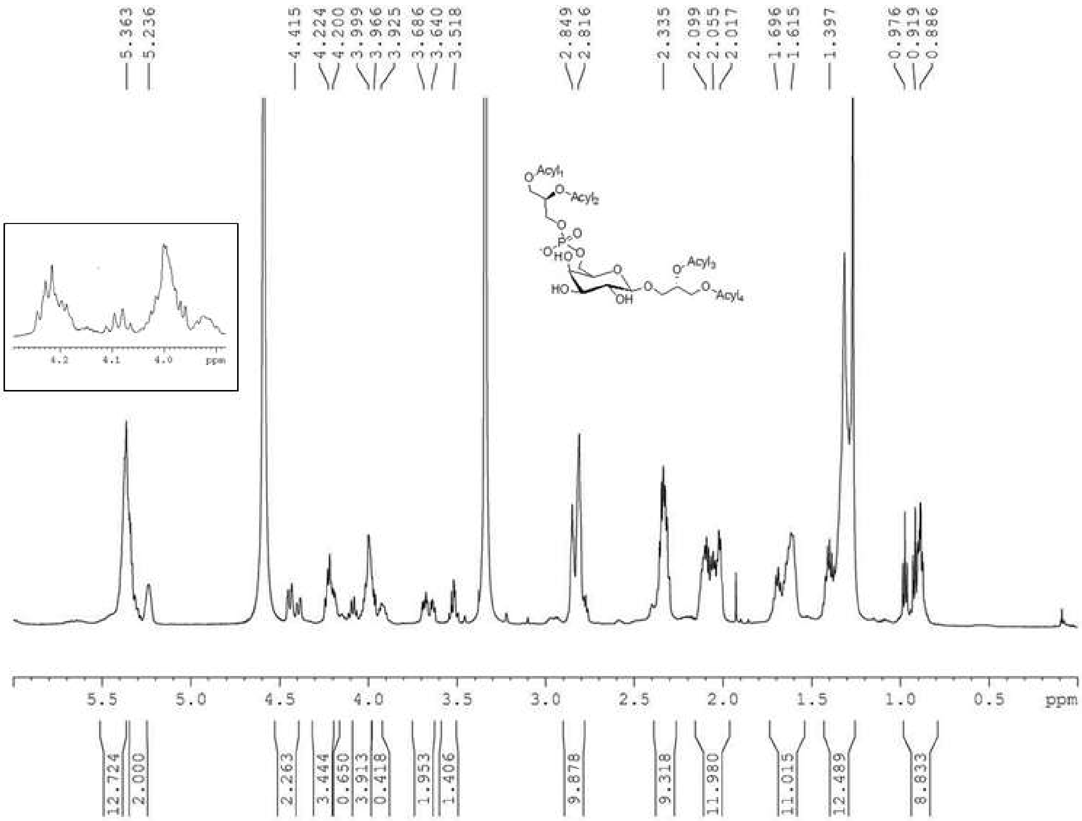

Figure S2. ^{13}C NMR spectrum of 1 (600MHz, $CDCl_3$/MeOD, 1:1).

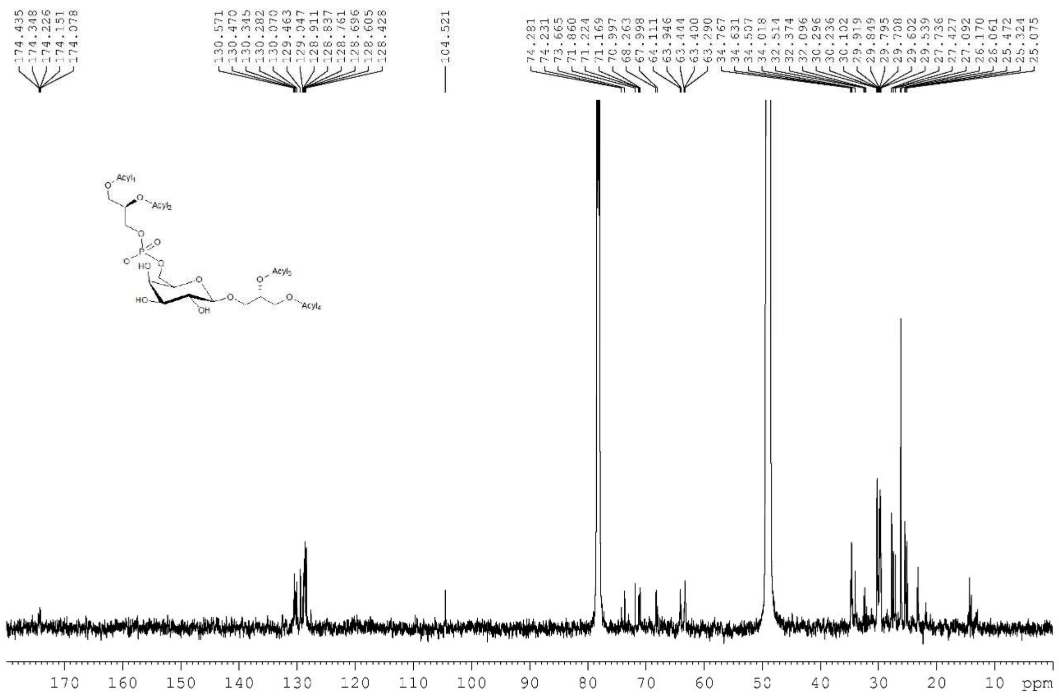

Figure S3. ^{1}H,^{1}H-COSY spectrum of 1 (600MHz, CDCl₃/MeOD, 1:1).

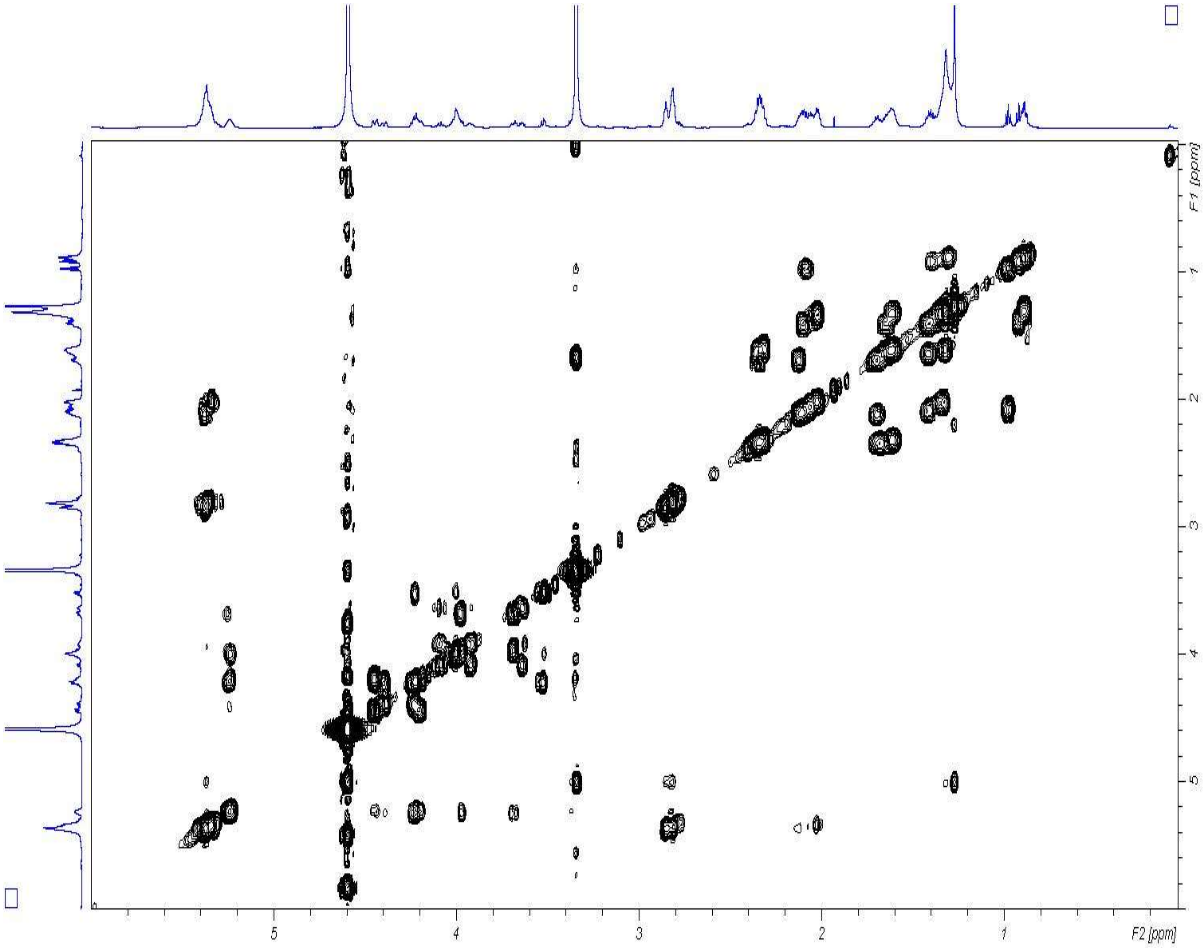

Figure S4. $^{1}H,^{1}H$-TOCSY spectrum of 1 (600MHz, $CDCl_3$/MeOD, 1:1).

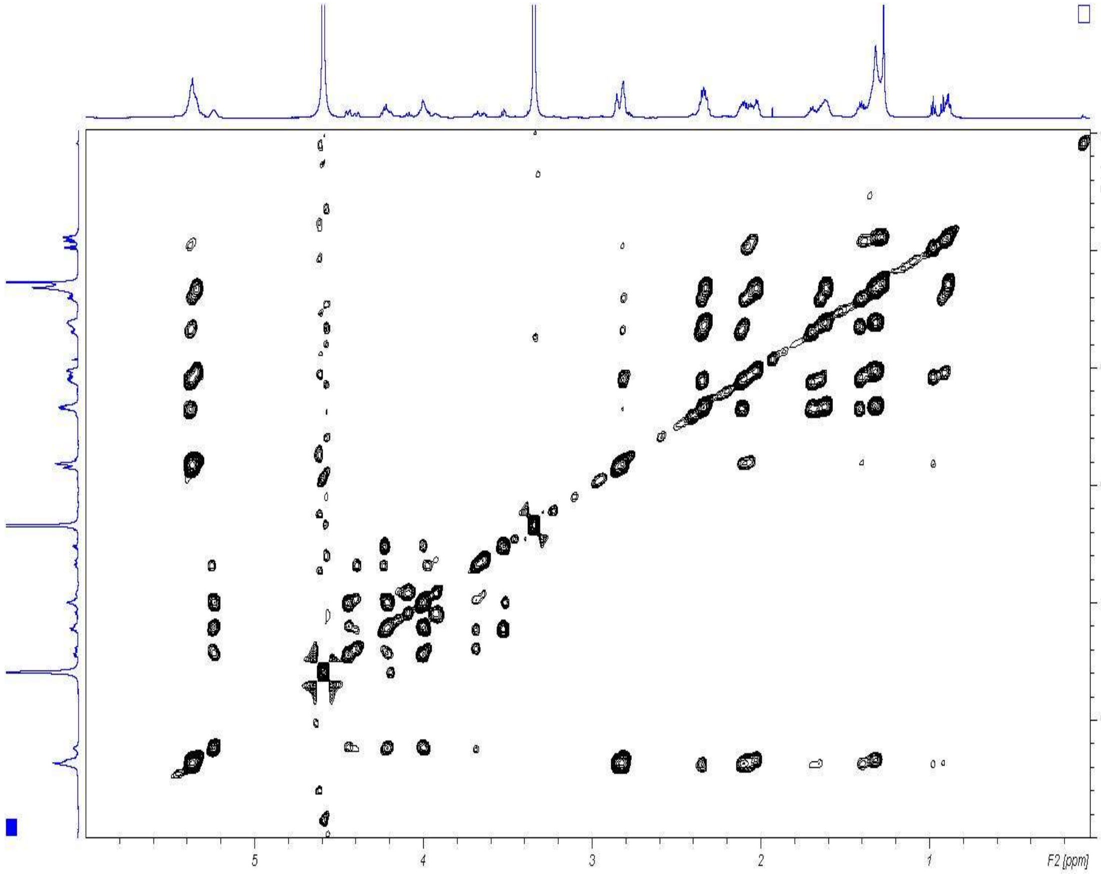

Figure S5. HSQC spectrum of 1 (600MHz, $CDCl_3$/MeOD, 1:1).

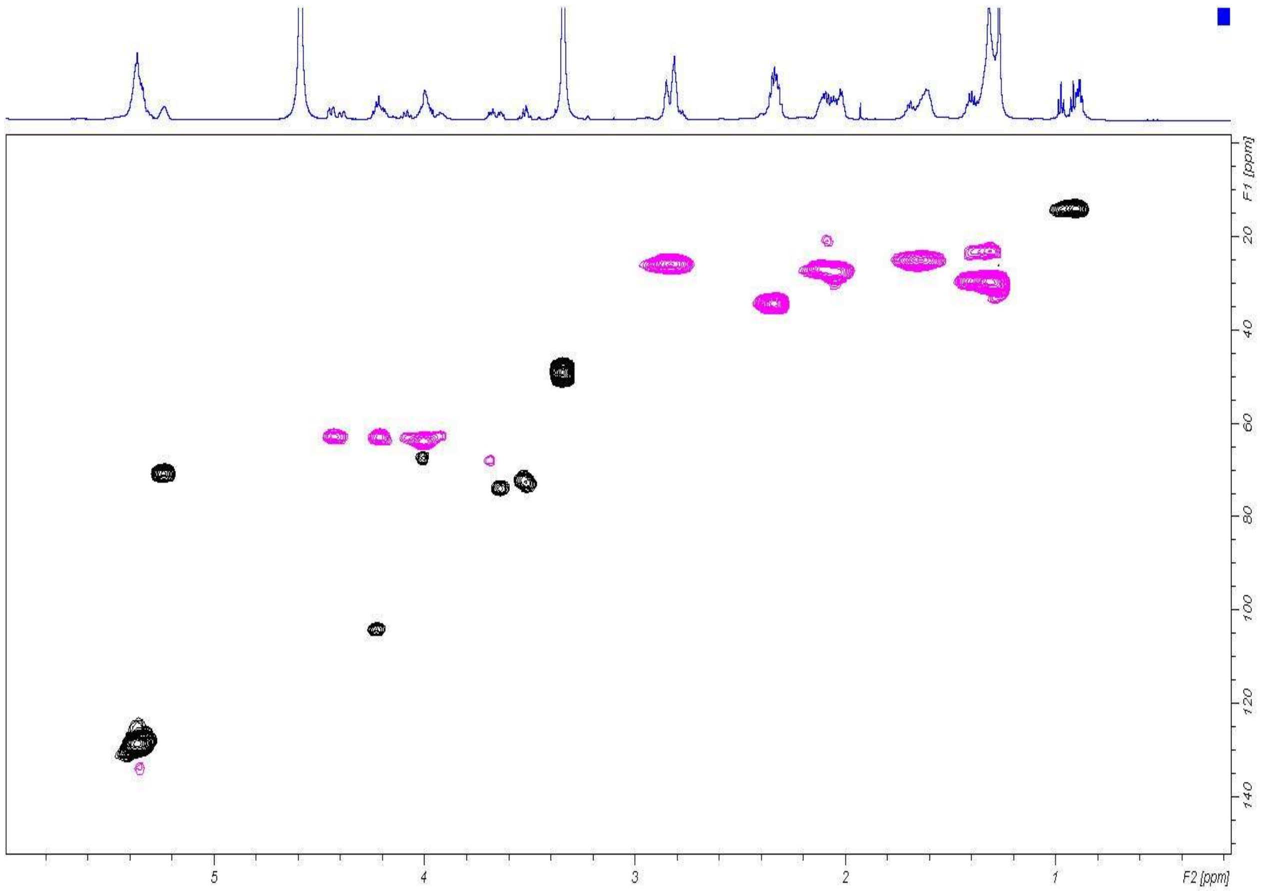

Figure S6. HMBC spectrum of 1 (600MHz, $CDCl_3$/MeOD, 1:1).

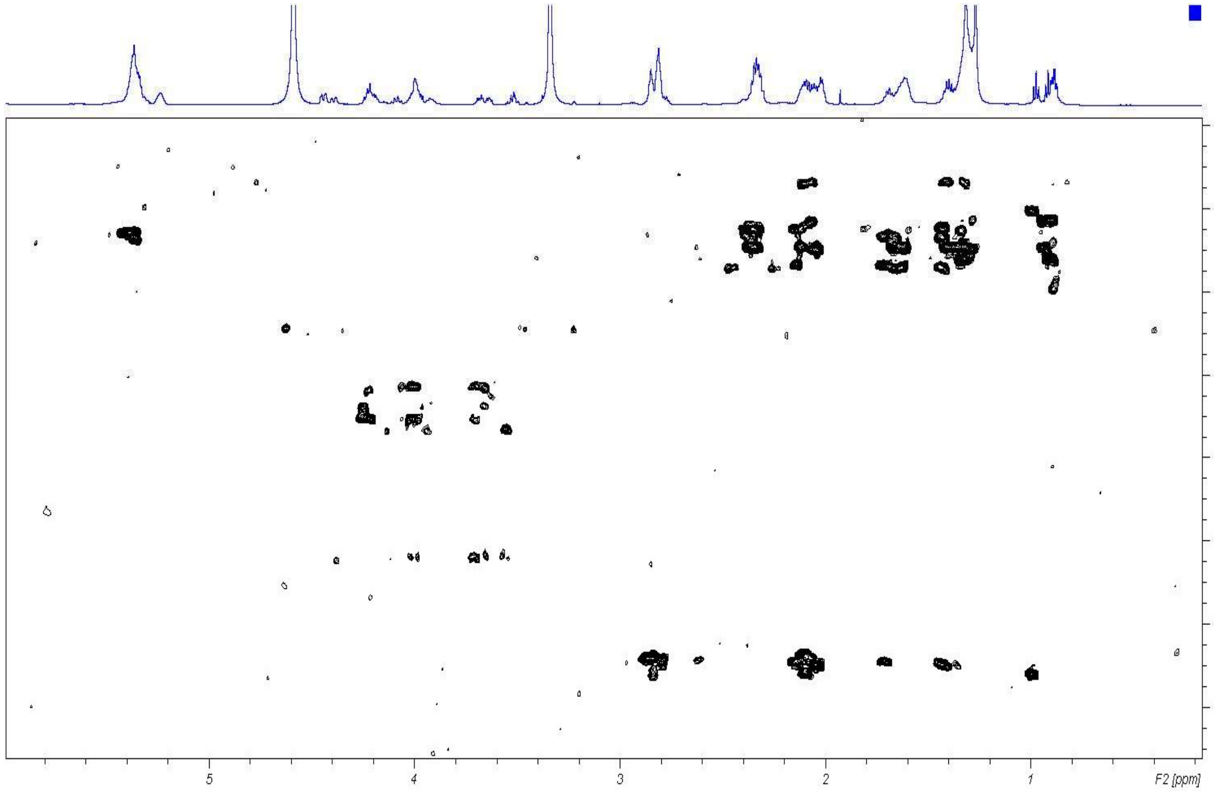

Figure S7. ^{1}H **NMR spectrum of 2 (600MHz,** $CDCl_3$**/MeOD, 1:1).**

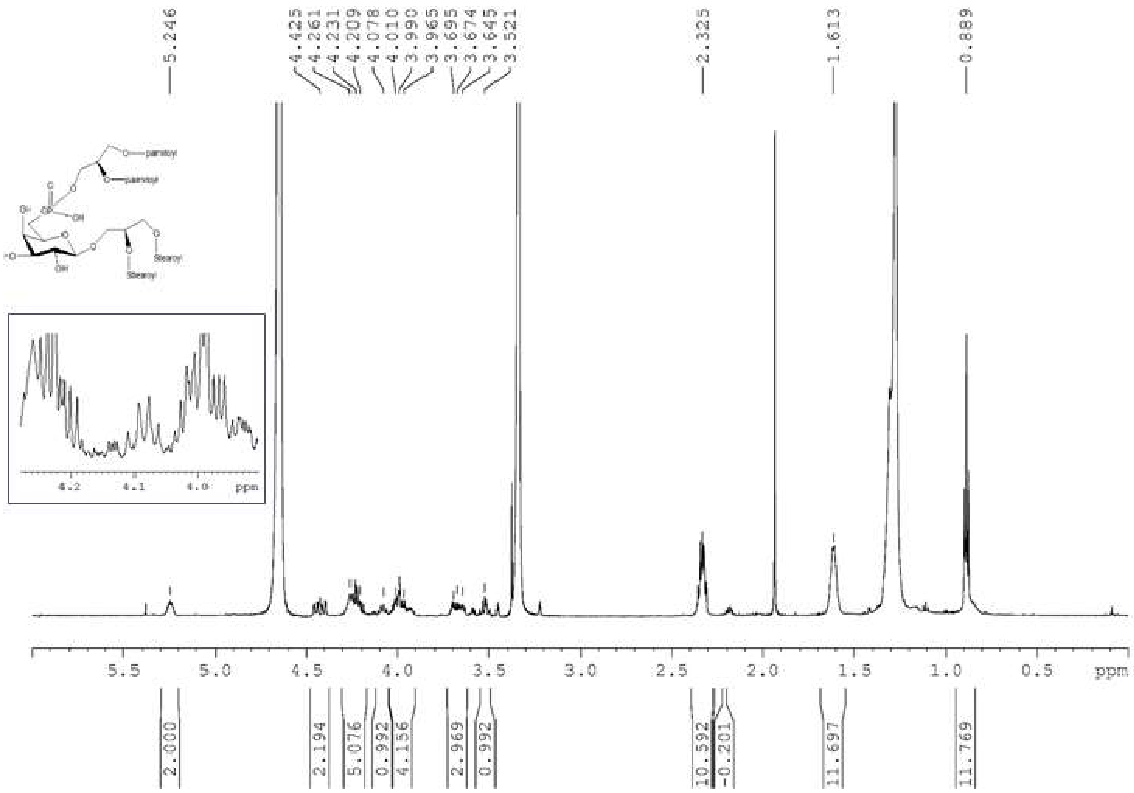

Figure S8. ^{13}C NMR spectrum of 2 (600MHz, $CDCl_3$/MeOD, 1:1).

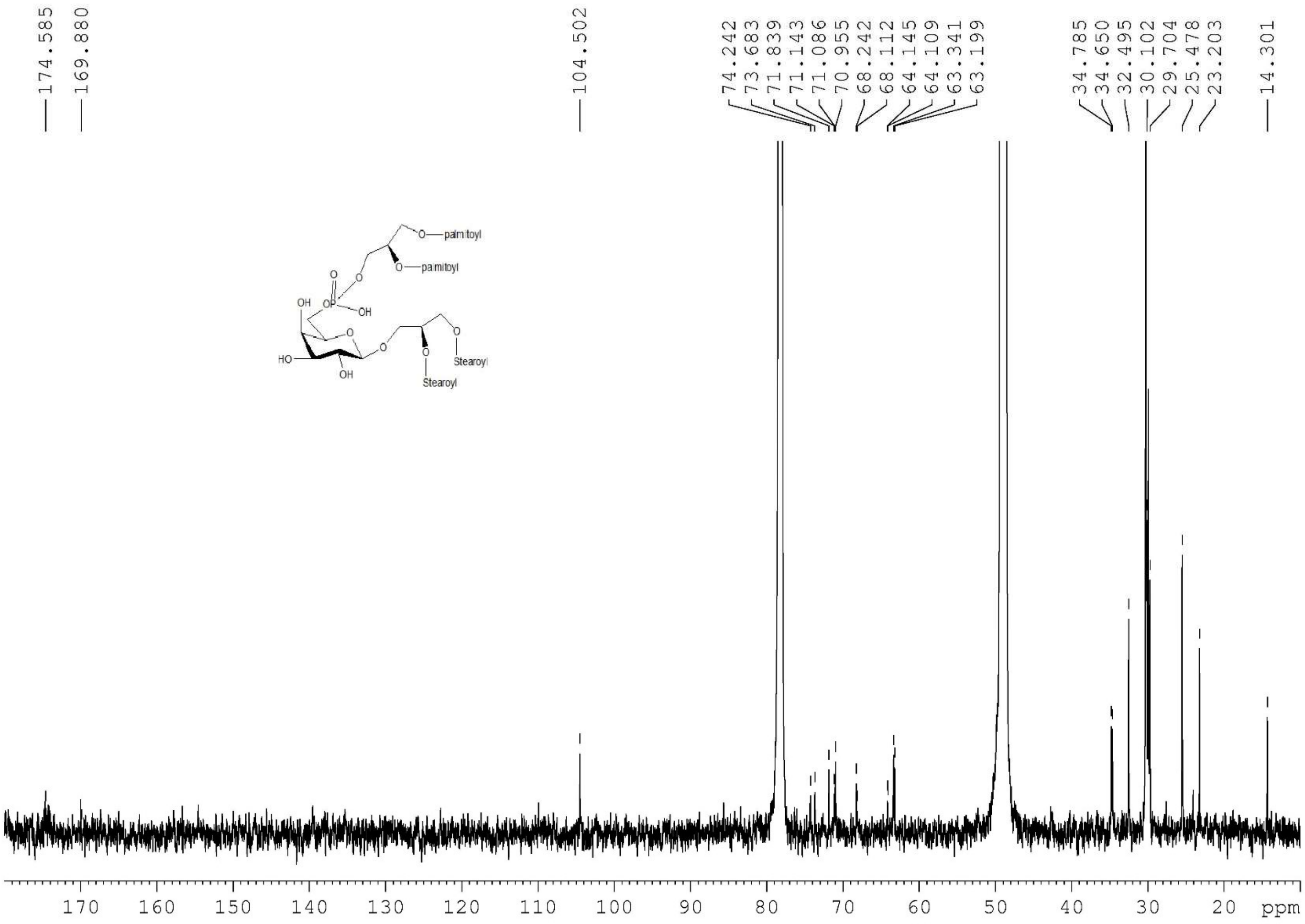

Figure S9. ^{1}H NMR spectrum of intermediate V (400MHz, CDCl₃).

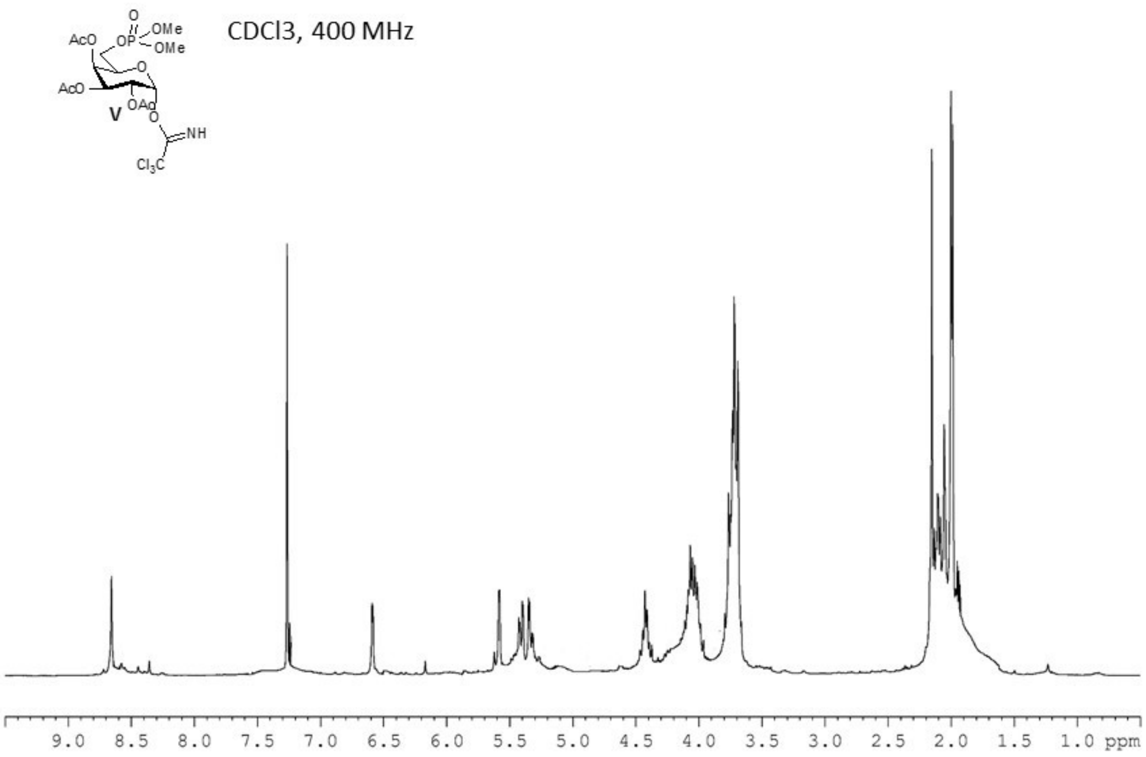

Figure S10. ^{1}H NMR spectrum of intermediate VI (400MHz, CDCl3).

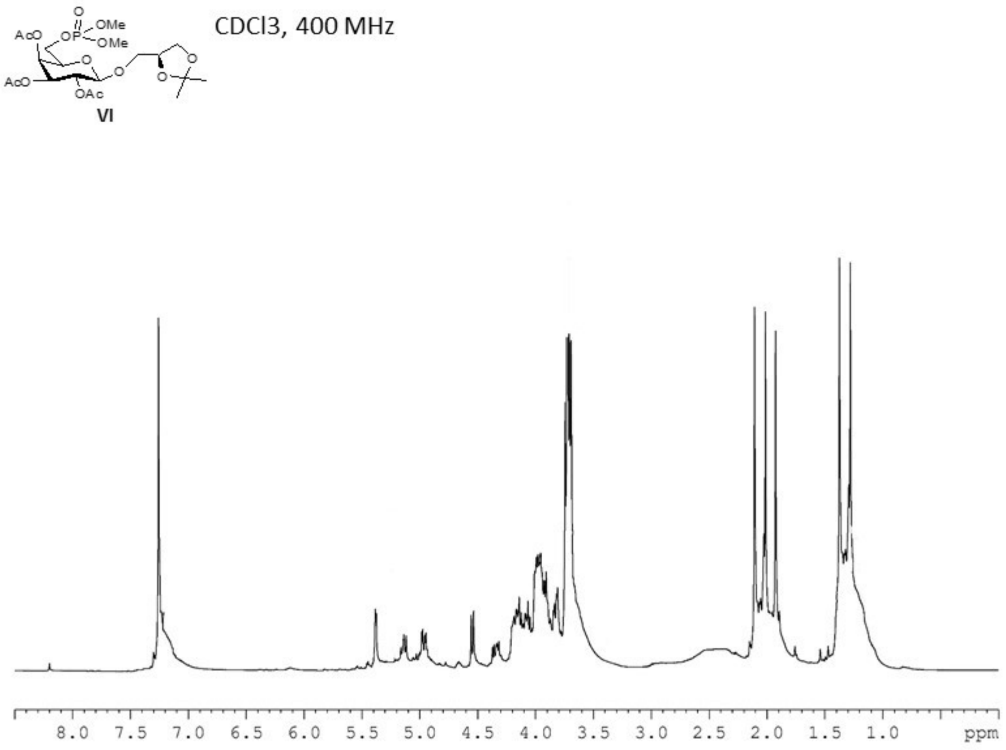

Figure S11. ^{1}H NMR spectrum of intermediate VII (400MHz, $CDCl_3$).

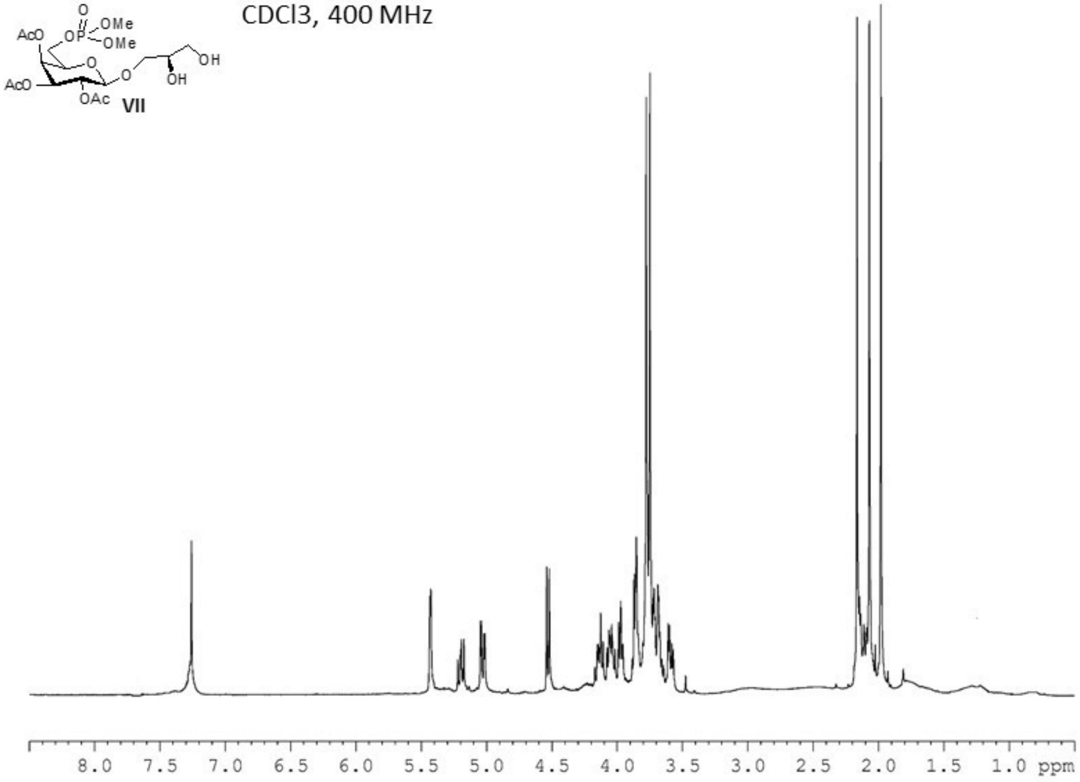

Figure S12. ^{1}H NMR spectrum of intermediate VIII (400MHz, CDCl3).

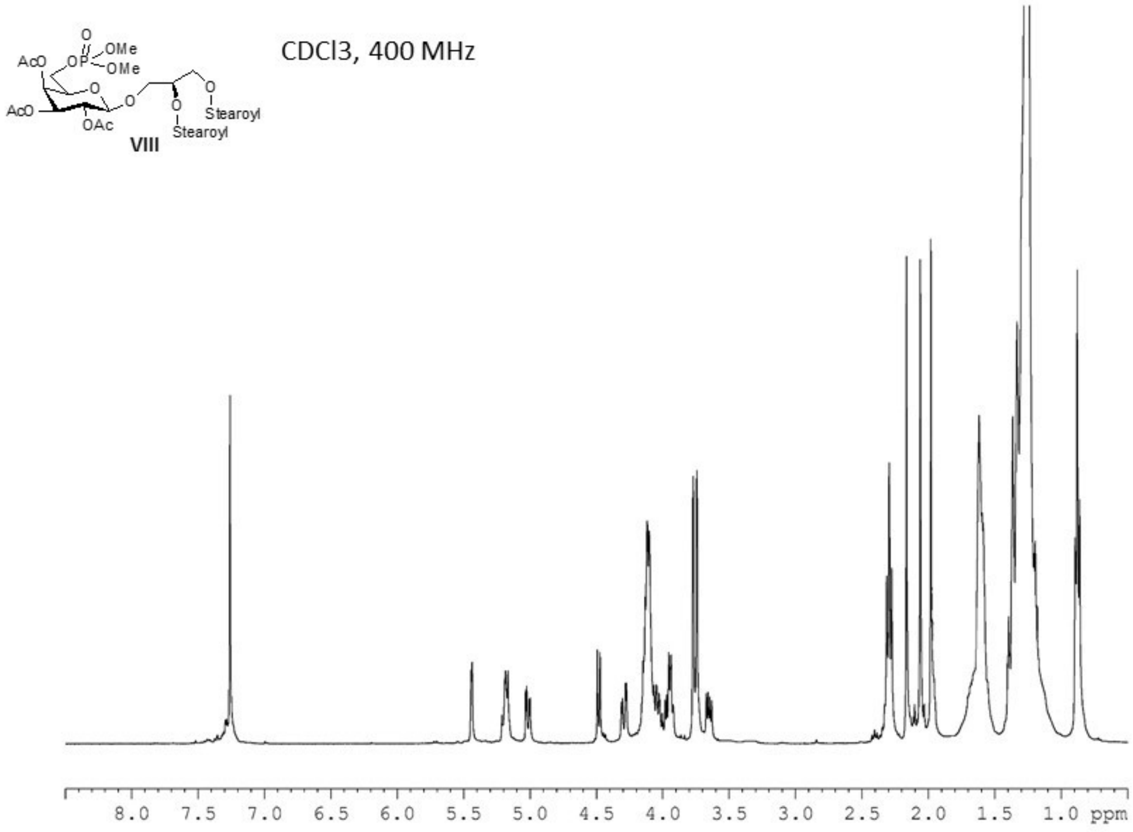

Figure S13. ^{1}H NMR spectrum of intermediate IX (400MHz, $CDCl_3$).

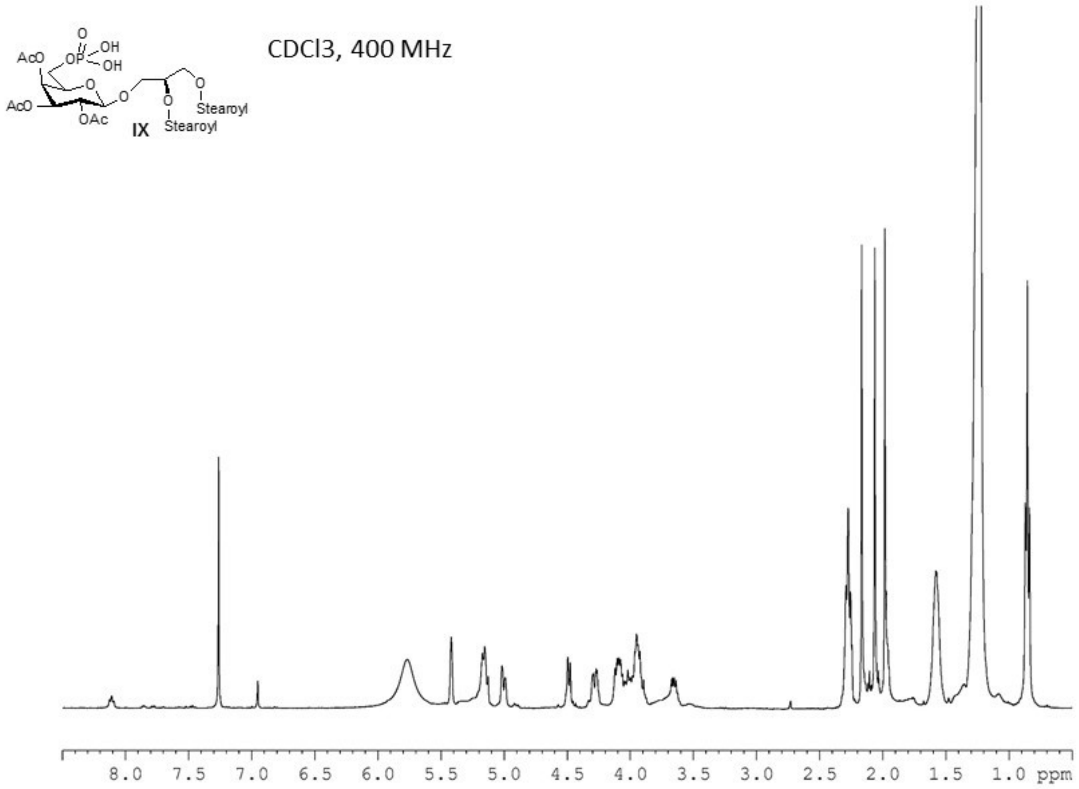

Figure S14. ¹H NMR spectrum of intermediate X (400MHz, MeOD).

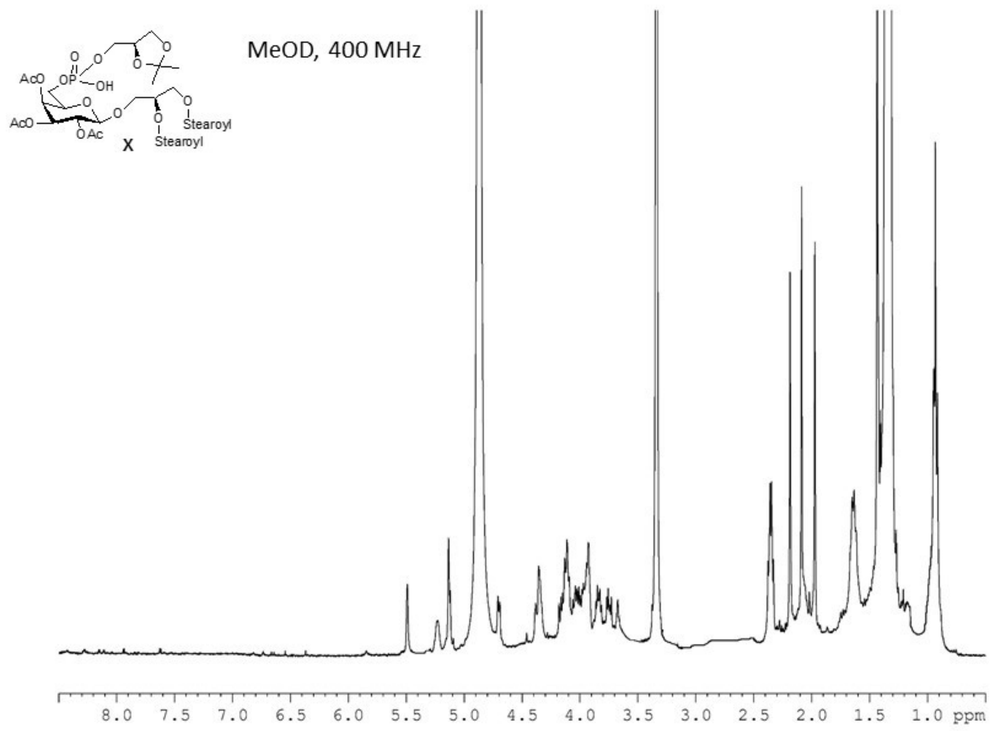

Figure S15. ^{1}H NMR spectrum of intermediate XI (400MHz, CDCl₃).

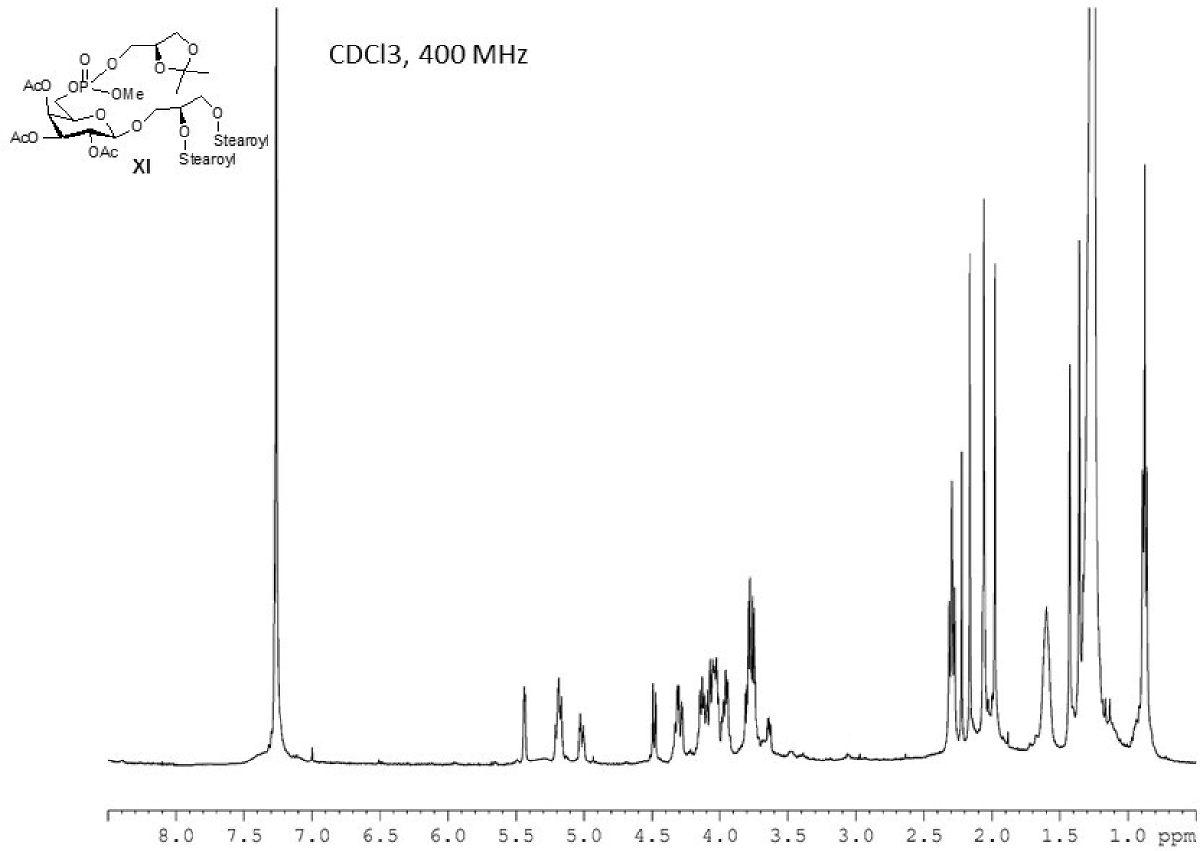

Figure S16. ^{1}H NMR spectrum of intermediate XII (400MHz, $CDCl_3$).

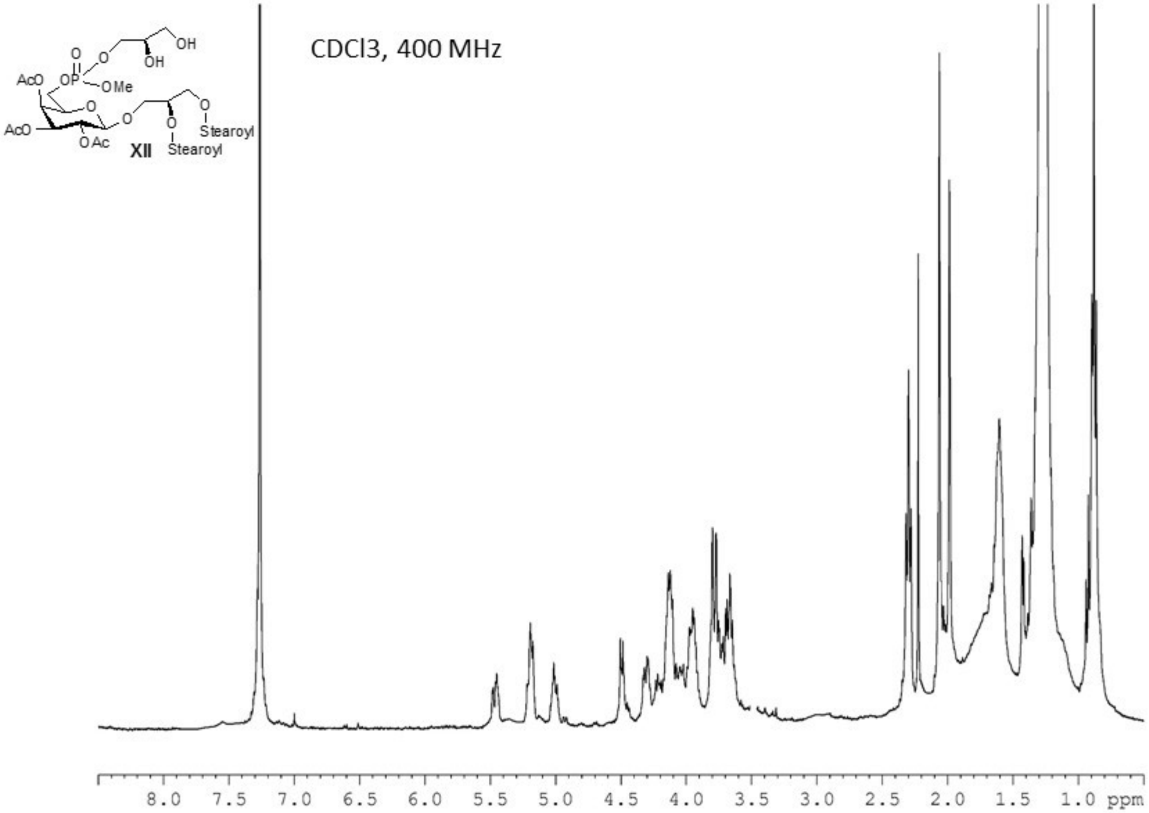

Figure S17. ^{1}H NMR spectrum of intermediate XIII (400MHz, $CDCl_3$).

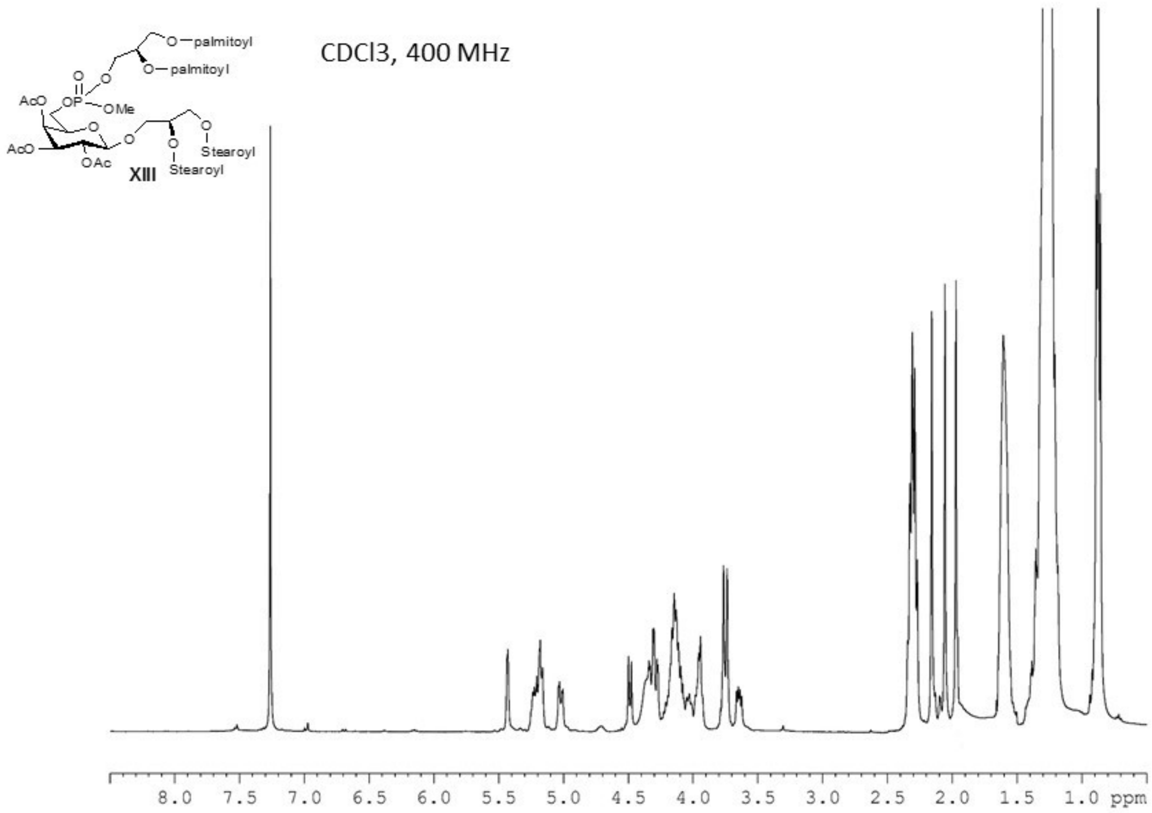

Figure S18. ^{1}H NMR spectrum of intermediate XIV (400MHz, $CDCl_3$)

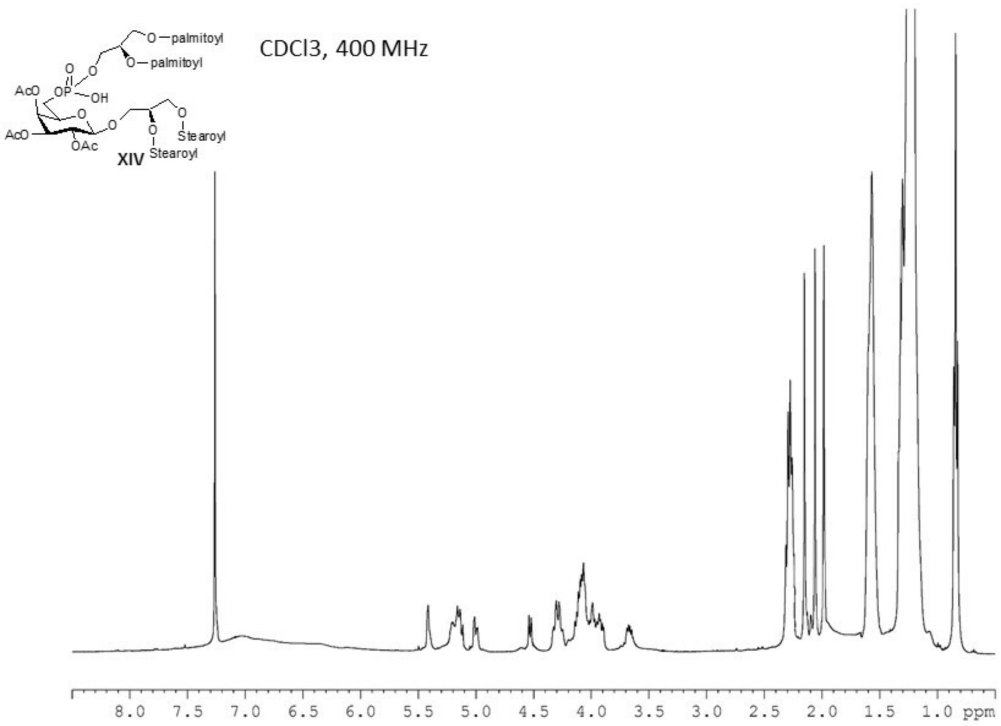

Figure S19. HR-ESI-MS spectrum of bioactive lipid pool isolated from the marine diatom *T. weissflogii*.

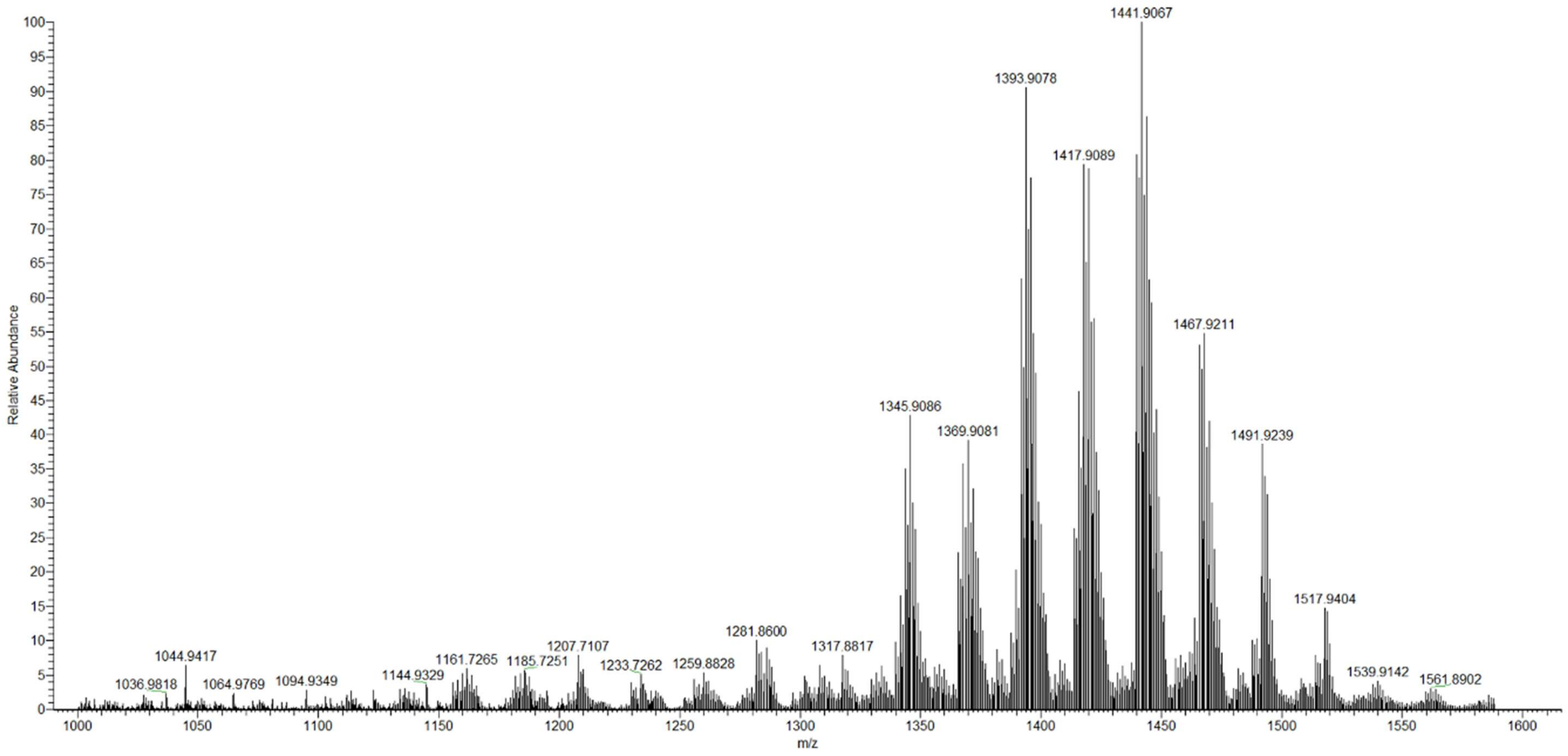

Table S1. Fatty acid methyl ester relative composition by GC-MS analysis.

Fatty acid	% Area
C14:0	1.6
C16:3	16.1
C16:1+C16:2	38.7
C16:0	17.9
C18:2	4.1
C18:1	3.1
C18:0	3.2
C20:5	14.1
C22:6	1.3

Table S2. Fatty acid composition of main species of the natural pool 1 by LC-ESI⁻/MS/MS analysis

C:N	Measured *m/z*	Calculated *m/z*	Formula	MS/MS (m/z)	FA composition
64:10	1339.8646	1339.8582	$C_{76}H_{124}O_{17}P^-$	253.2180, 249.1867	3C16:3, C16:1
64.9	1341.8786	1341.8738	$C_{76}H_{126}O_{17}P^-$	253.2171, 251.2020, 249.1858	2C16:3, C16:2, C16:1
64:8	1343.8945	1343.8895	$C_{76}H_{128}O_{17}P^-$	253.2179, 249.1852	2C16:1, 2C16:3
64:7	1345.9086	1345.9051	$C_{76}H_{130}O_{17}P^-$	251.2020, 253.2176, 255.2321	C16:3, 2C16:2, C16:0
64:6	1347.9230	1347.9208	$C_{76}H_{132}O_{17}P^-$	253.2174, 251.2013	2C16:2, 2C16:1
66:9	1369.9081	1369.9051	$C_{78}H_{130}O_{17}P^-$	253.2184, 279.2351	C16:1, 2C16:3, C18:2
66:9	1369.9081	1369.9051	$C_{78}H_{130}O_{17}P^-$	227.2014, 249.1865, 277.2166	C14:0, C16:3, 2C18:3
66:8	1371.9229	1371.9208	$C_{78}H_{132}O_{17}P^-$	253.2174, 279.2326	C16:1, C16:2, C16:3, C18:2
66:8	1371.9229	1371.9208	$C_{78}H_{132}O_{17}P^-$	251.2013, 255.2335, 277.2177	C16:0, C16:2, C16:3, C18:3
66:8	1371.9229	1371.9208	$C_{78}H_{132}O_{17}P^-$	251.2013, 281.2485, 253.2174	C18:1, C16:3, 2C16:2
68:12	1391.8937	1391.8895	$C_{80}H_{128}O_{17}P^-$	253.2175, 249.1865, 301.2162	C20:5, C16:1, 2C16:3
68:11	1393.9078	1393.9051	$C_{80}H_{130}O_{17}P^-$	253.2174, 301.2173, 251.2020, 249.1853	C20:5, C16:1, C16:2, C16:3
68:11	1393.9078	1393.9051	$C_{80}H_{130}O_{17}P^-$	255.2329, 249.1865, 301.2180	C20:5, C16:0, 2C16:3
68:10	1395.9222	1395.9208	$C_{80}H_{132}O_{17}P^-$	253.2174, 251.2020, 301.2173	C20:5, 2C16:1, C16:2
68:10	1395.9222	1395.9208	$C_{80}H_{132}O_{17}P^-$	301.2175, 251.2020, 255.2326	C20:5, C16:0, 2C16:2
68:9	1397.9369	1397.9364	$C_{80}H_{134}O_{17}P^-$	253.2180, 277.2166	2C18:3, C16:1, C16:2
70:14	1415.8913	1415.8895	$C_{82}H_{128}O_{17}P^-$	277.2173, 301.2165, 251.2013	C16:4, C16:2, C18:3, C20:5
70.13	1417.9089	1417.9051	$C_{82}H_{130}O_{17}P^-$	279.2334, 301.2162, 249.1867	2C16:3, C18:2, C20:5
70:12	1419.9233	1419.9208	$C_{82}H_{132}O_{17}P^-$	279.2330, 249.1852, 301.2162, 250.9681	C16:3, C16:2, C18:2, C20:5
70:11	1421.9377	1421.9364	$C_{82}H_{134}O_{17}P^-$	279.2351, 253.2175	C16:3, C16:1, C18:2, C20:5
72:16	1439.8933	1439.8895	$C_{84}H_{128}O_{17}P^-$	301.2173, 249.1852	2C20:5, 2C16:3
72:15	1441.9067	1441.9051	$C_{84}H_{130}O_{17}P^-$	301.2173, 251.2005	2C20:5, C16:2, C16:3
72:14	1443.9220	1443.9208	$C_{84}H_{132}O_{17}P^-$	301.2165, 253.2175	2C20:5, C16:1, C16:3
74:17	1465.908	1465.9051	$C_{86}H_{130}O_{17}P^-$	275.5774, 301.2183, 249.1852	2 C20:5, C18:4, C16:3
74.16	1467.9211	1467.9208	$C_{86}H_{132}O_{17}P^-$	301.2176, 277.2172	2C20:5, C18:3, C16:3
74:15	1469.9211	1469.9364	$C_{86}H_{134}O_{17}P^-$	301.2133, 279.2332	2C20:5, C18:2, C16:3
76:18	1491.9239	1491.9208	$C_{88}H_{132}O_{17}P^-$	301.2173	3C20:5, C16:3
78:19	1517.9404	1517.9364	$C_{90}H_{134}O_{17}P^-$	301.2166	3C20:5, C18:4

Figure S20. Expression of cell surface markers in MoDCs by stimulation with LH-20 bioactive fraction from *T. weissflogii.*

Expression of CD83, CD86 and HLA-DR by MoDCs stimulated with LH-20 bioactive fraction from the extract of *T. weissflogii* in the concentration range 10 -50 µg/mL.

Results (mean +/− standard deviation) are reported as percentage of positive cells (for HLA-DR marker we considered an expression value over 10^4) out of the total DC population. Asterisks indicate significant differences from the cells treated only with vehicle (control, Ctrl) at a 95% ($P < 0.05$) confidence level, as determined using one-way ANOVA analysis; **** $p < 0.0001$.

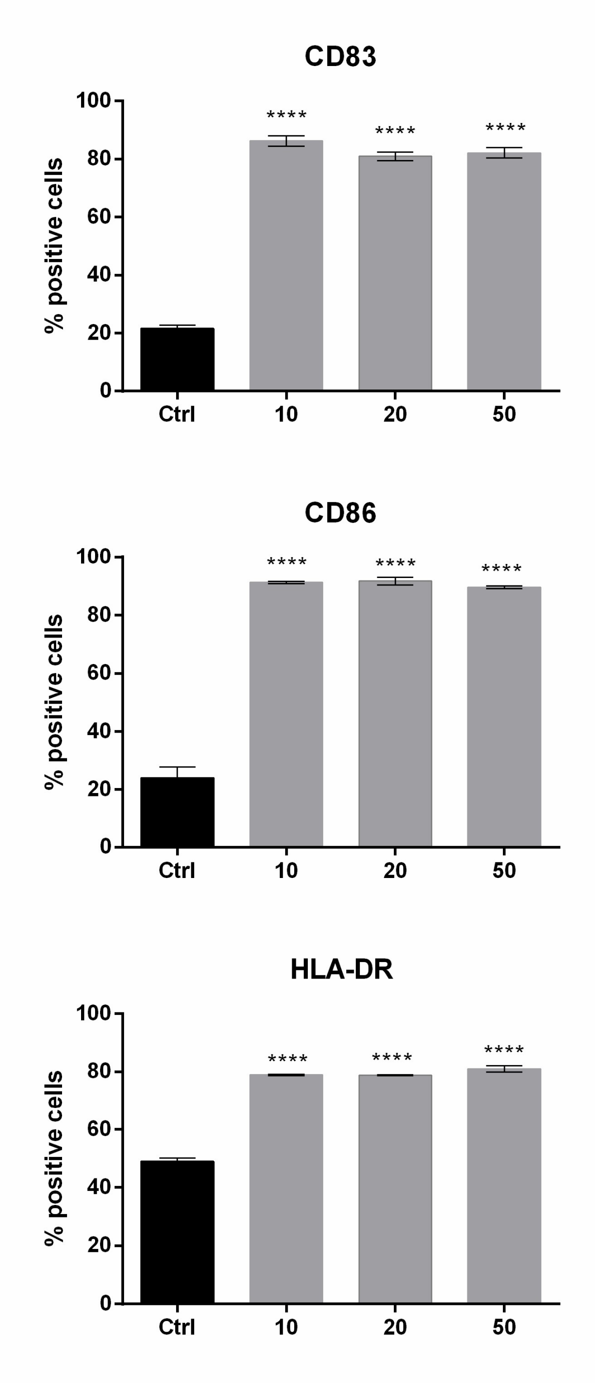

Supplement: Supplementary file 1 [file marinedrugs-17-00103-s001.pdf]
